# Supplementary material for: Alignstein: Optimal transport for improved LC-MS retention time alignment
Source: Gigascience. 2022 Nov 3;11:giac101. doi: 10.1093/gigascience/giac101 (PMC9633278; doi:10.1093/gigascience/giac101)
Supplement: giac101_GIGA-D-22-00149_Revision_2 [file giac101_giga-d-22-00149_revision_2.pdf]

GigaScience

Alignstein: optimal transport for improved LC-MS retention time alignment

--Manuscript Draft--

Manuscript Number:	GIGA-D-22-00149R2
Full Title:	Alignstein: optimal transport for improved LC-MS retention time alignment
Article Type:	Technical Note
Funding Information:	Narodowe Centrum Nauki (2019/33/N/ST6/02949) — Mr. Grzegorz Skoraczyński<br>Narodowe Centrum Nauki (2018/29/B/ST6/00681) — prof. Anna Gambin, prof. Błażej Miasojedow
Abstract:	Background<br>Reproducibility of liquid chromatography separation is limited by retention time drift. As a result, measured signals lack correspondence over replicates of the liquid chromatography mass spectrometry (LC-MS) experiments. Correction of these errors is named retention time alignment and needs to be performed before further quantitative analysis. Despite the availability of numerous alignment algorithms, their accuracy is limited, e.g. for retention time drift that swaps analytes' elution order.<br>Results<br>We present the Alignstein, an algorithm for liquid chromatography-mass spectrometry retention time alignment. It correctly finds correspondence even for swapped signals. To achieve this, we implemented the generalization of the Wasserstein distance to compare multidimensional features without any reduction of the information or dimension of the analyzed data. Moreover, Alignstein by design requires neither a reference sample nor prior signal identification. We validate the algorithm on publicly available benchmark datasets obtaining competitive results. Finally, we show that it can detect the information contained in the tandem mass spectrum by the spatial properties of chromatograms.<br>Conclusions<br>We show that the use of optimal transport effectively overcome the limitations of existing algorithms for statistical analysis of mass spectrometry datasets. The algorithm's source code is available at https://github.com/grzsko/Alignstein.
Corresponding Author:	Grzegorz Skoraczyński, M.S.<br>University of Warsaw Faculty of Mathematics Informatics and Mechanics: Uniwersytet Warszawski Wydzial Matematyki Informatyki i Mechaniki<br>Warsaw, POLAND
Corresponding Author Secondary Information:	
Corresponding Author's Institution:	University of Warsaw Faculty of Mathematics Informatics and Mechanics: Uniwersytet Warszawski Wydzial Matematyki Informatyki i Mechaniki
Corresponding Author's Secondary Institution:	
First Author:	Grzegorz Skoraczyński, M.S.
First Author Secondary Information:	
Order of Authors:	Grzegorz Skoraczyński, M.S.<br>Anna Gambin, professor<br>Błażej Miasojedow, professor
Order of Authors Secondary Information:	
Response to Reviewers:	Dear Editor,<br><br>We have made all the formatting changes you requested.<br><br>Grzegorz Skoraczyński, Anna Gambin, Błażej Miasojedow

Powered by Editorial Manager® and ProduXion Manager® from Aries Systems Corporation

Additional Information:	
**Question**	**Response**
Are you submitting this manuscript to a special series or article collection?	No
**Experimental design and statistics**<br><br>Full details of the experimental design and statistical methods used should be given in the Methods section, as detailed in our Minimum Standards Reporting Checklist. Information essential to interpreting the data presented should be made available in the figure legends.<br><br>Have you included all the information requested in your manuscript?	Yes
**Resources**<br><br>A description of all resources used, including antibodies, cell lines, animals and software tools, with enough information to allow them to be uniquely identified, should be included in the Methods section. Authors are strongly encouraged to cite Research Resource Identifiers (RRIDs) for antibodies, model organisms and tools, where possible.<br><br>Have you included the information requested as detailed in our Minimum Standards Reporting Checklist?	Yes
**Availability of data and materials**<br><br>All datasets and code on which the conclusions of the paper rely must be either included in your submission or deposited in publicly available repositories (where available and ethically appropriate), referencing such data using a unique identifier in the references and in the "Availability of Data and Materials" section of your manuscript.	Yes

Powered by Editorial Manager® and ProduXion Manager® from Aries Systems Corporation

Have you have met the above requirement as detailed in our Minimum Standards Reporting Checklist?	

Manuscript Click here to access/download;Manuscript;main.tex

OXFORD (GIGA)^n SCIENCE

GigaScience, 2017, 1–9

doi: xx.xxxx/xxxx
Manuscript in Preparation
Technical note

TECHNICAL NOTE

Alignstein: optimal transport for improved LC-MS retention time alignment

Grzegorz Skoraczyński[1,*], Anna Gambin[1] and Błażej Miasojedow[1]

[1]Faculty of Mathematics, Informatics, and Mechanics, University of Warsaw, Stefana Banacha 2, 02-097 Warsaw, Poland

[*] Corresponding author. E-mail: g.skoraczynski@mimuw.edu.pl

Abstract

Background Reproducibility of liquid chromatography separation is limited by retention time drift. As a result, measured signals lack correspondence over replicates of the liquid chromatography mass spectrometry (LC-MS) experiments. Correction of these errors is named retention time alignment and needs to be performed before further quantitative analysis. Despite the availability of numerous alignment algorithms, their accuracy is limited, e.g. for retention time drift that swaps analytes' elution order.
Results We present the Alignstein, an algorithm for liquid chromatography-mass spectrometry retention time alignment. It correctly finds correspondence even for swapped signals. To achieve this, we implemented the generalization of the Wasserstein distance to compare multidimensional features without any reduction of the information or dimension of the analyzed data. Moreover, Alignstein by design requires neither a reference sample nor prior signal identification. We validate the algorithm on publicly available benchmark datasets obtaining competitive results. Finally, we show that it can detect the information contained in the tandem mass spectrum by the spatial properties of chromatograms.
Conclusions We show that the use of optimal transport effectively overcome the limitations of existing algorithms for statistical analysis of mass spectrometry datasets. The algorithm's source code is available at
https://github.com/grzsko/Alignstein.

Key words: liquid chromatography-mass spectrometry; retention time alignment; Wasserstein distance; simplex algorithm

Introduction

Advances in liquid chromatography mass spectrometry (LC-MS) have provided a remarkable insight into the functioning of the organisms, ranging from protein level [1], through tissue [2] to environmental networks [3]. All of these research studies benefit from the possibility to separate complex mixtures in the liquid chromatographic column and then measure the analytes with high throughput mass spectrometer. Although LC-MS systems provide precise answers to both quantitative and qualitative biological and medical questions, designing algorithms for efficient and precise analysis of LC-MS datasets remains challenging.

One of these challenges is the correction of errors caused by retention time (RT) drift. It limits the reproducibility of LC separation, which is important for experiments usually acquired in many (even hundreds) replicates. RT drift became a significant obstacle with the emergence of high-performance chromatography (HPLC) and ultra-performance chromatography (UPLC) technologies. For example, nanoflow UPLC column separation takes a relatively long time, usually up to several hours. For these experiments, the elution time of peptides may vary up to 5 minutes [4] or even 10 minutes [1].

RT drift can be corrected by the experimental protocol only to a limited extent [5]. It may change the whole gradient or affect only single peaks. These changes may be caused by various reasons such as the unstable mobile phase, the column

Compiled on: September 28, 2022.
Draft manuscript prepared by the author.

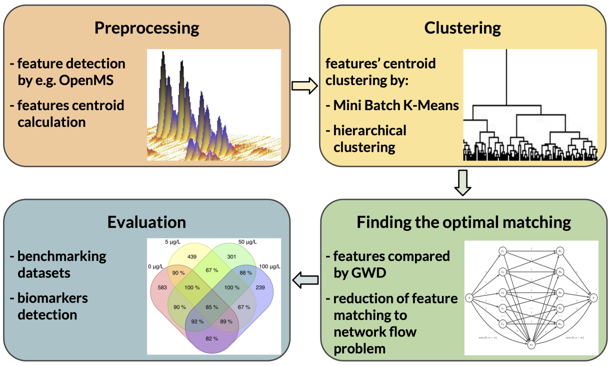

Figure 1. The outline of the Alignstein algorithm. It starts with feature preprocessing, for which then centroids are computed and clustered. As a next step, the problem of optimal feature matching is solved. The result is obtained with regard to prior clustering and can be further analyzed and verified.

change or degradation, sample chemical instability, or imprecise experiment setup [6, 7, 8].

RT drift requires a correction, usually named the RT alignment. It results in the correspondence of signals across runs [9]. For example, in proteomics, the signal correspondence of the same peptides is needed for further applying label-free quantification (LFQ) for which samples must be measured separately [10, 11]. Moreover, for LFQ techniques, we cannot obtain the correspondence any other way because analytes do not have any additional information, such as metabolic labels, or chemical tags [12, 13].

Here, we present a novel alignment algorithm named Alignstein (cf. Fig. 1). It finds the correspondence of initially detected features, i.e. convex sets of peaks representing the signal of a single analyte. It overcomes the limitations of currently existing algorithms and properly resolves the correspondence of analytes of swapped elution order. To achieve this, we take advantage of the generalization of the Wasserstein distance (GWD) [14] to compare multidimensional features. To obtain the most feasible alignment results, Alignstein has formulated a complex optimization signal-matching problem, for which we use clustering and network flow algorithms to achieve a computationally tractable outcome.

This article is organized as follows. First, we characterize Alignstein and analyze how it deals with the swapped signals. Then, we validate the algorithm on publicly available benchmark datasets. Finally, we show the applicability of our approach to detecting corresponding biomarkers in differing samples.

Findings

The problem: resolving swaps

RT drift may swap the order of eluting analytes. In the proteomic experiment (cf. Methods), we analyzed that about 3 % of all feature pairs are swapped between two chromatograms. Although many of the available algorithms properly align most signals, still they fail to resolve swaps.

The vast majority of approaches to RT alignment are so-called warping algorithms, e.g. OpenMS [15], MetAlign [16], MZMine 2 [17], SIMA [18], the solution proposed by Zhang [19], DIAlignR [20], the solution proposed by Chiung-Ting Wu, et al. [21]. These algorithms consist of applying a warping function that transforms the chromatograms by shifting, stretching, and squeezing. These transformations result in a close distance between corresponding signals. After alignment, however, further feature detection and matching are still required to obtain the signal correspondence. These algorithms' applicability is limited because the warping function is applied under the assumption that ions elute monotonically with RT. Thus, they are not able to deal with elution order swaps.

Alternatively, a rarer implemented approach is feature matching, e.g. OpenMS [15] (both warping and matching algorithm), MassUntangler [22], LWBMatch [23], the solution proposed by Wandy, et al. [24], MS-Dial [25], Quandenser [26]. Algorithms by feature matching find the correspondence between initially detected features of two or more chromatograms. Corresponding features represent the same analyte and further will be referred to as consensus features. To the best of the authors' knowledge, all matching algorithms reduce multidimensional features to one-dimensional extracted ion chromatograms or a single points with monoisotopic peak M/Z and average RT value, ignoring the information of isotopic envelope or feature span over the RT dimension. Without feature spatial characteristics and information of coeluting ions, elution order swaps are practically undetectable [8]. The main reason for this simplification lies in the difficulty to find multidimensional feature dissimilarity measures. Typically, Euclidean distance between points or one-dimensional cosine-like spectra similarity scores is applied [27, 28]. Although the limitations of these scores are known, still there is a shortage of their effective improvements [28, 29].

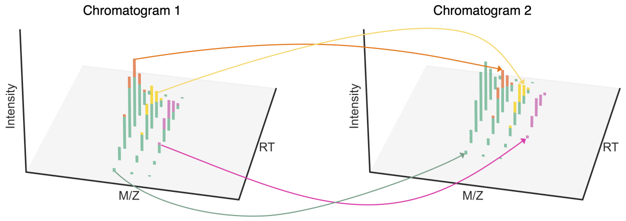

Figure 2. The optimal transport plan between two features. The Wasserstein distance captures not only the distance of feature drift along the RT dimension but also spatial differences between features. Here, the left feature consists of three ions, right feature consists of four ions. To properly capture this difference, part of the signal must be transported between different ions (denoted with arrows) and thus the transport cost (the Wasserstein distance) is higher.

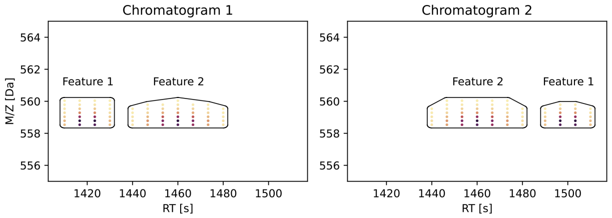

Figure 3. Example of swapped features. They represent four times charged peptides: HTALYSSDSVRNVRKKDTTG (Feature 1) and HTAIYSSDSVRNVRKKDTTG (Feature 2). Isotopic envelopes were generated using the IsoSpec tool [30] and smoothed over RT with a gaussian filter. Retention times were predicted using Pyteomics package [31]. The Euclidean distance between corresponding shifted features reduced to a point is 0.0 and 80.0, and between non-corresponding features is 40.0 and 40.0. Whereas GWD for corresponding features equals 0.3 and 80.3, and for non-corresponding features, to 46.3 and 46.3. For such an example, a simple feature matching algorithm, using GWD, would match the features correctly, and for the Euclidean distance, this solution would be ambiguous.

The solution: the Alignstein algorithm

Alignstein is the RT alignment algorithm by feature matching that properly deals with features of swapped order. It is possible because the algorithm represents features by all signals contained within their boundaries. To cope with this representation, we use the generalization of the Wasserstein distance as a feature dissimilarity measure. It originates from the optimal transport theory and has been recently attracting growing attention to various problems of mass spectrometry [27, 32, 33, 34, 35]. Its design significantly differs from currently existing similarity scores and thus it overcomes the majority of their limitations. The Wasserstein distance describes the cost of the optimal way how to transform one feature into the other one. The transformations include not only shifting the signal from one feature to another but also splitting or combining the signal between peaks (cf. Fig. 2). The key strength of Wasserstein distance is the ability to compute features' similarity by their spatial shape (cf. Fig. 3). Moreover, it easily scales with dimension. Generalizing the Wasserstein distance allows comparing noisy features by introducing an appropriate penalty. This provides a highly flexible measure for effective computing feature distance and similarity.

Alignstein aligns chromatograms by finding consensus features. It is done in two phases (cf. Fig. 1): at first, feature centroids are clustered to find candidates for consensus features, which are then verified by the feature matching phase. During the latter phase, the algorithm computes the optimal feature matching, which represents the most similar feature pairs throughout all chromatograms (cf. Methods). We solve this problem by reducing it to finding the maximum flow of minimum cost in an appropriate flow network (cf. Fig. 4). Consensus features are then created from optimal feature matching with regard to initial centroid clustering. The such formulation allows for aligning chromatograms without a requirement for a reference sample or a prior feature identification. It also easily scales with a number of input chromatograms. Finally, this algorithm is not limited to correcting RT perturbations in repeated experimental runs, it also accurately aligns the majority of detected corresponding biomarkers from samples of different experimental treatments.

Dealing with swapped signal

We assessed that Alignstein properly matches swapped features. For this purpose, we collected over 580 identified features from the chromatograms obtained from Barranger et al.'s work [3] (see Methods). We simulated RT drift by randomly moving features within range (-150 s, 150 s) in the RT dimension and within range (-0.3 Da, 0.3 Da) in the M/Z dimension. These two sets of features: one with original features and the second with drifted features represented chromatograms to be aligned. For such a formulation, about 2 % (ca. 3400) of feature pairs were swapped. We aligned these two sets and measured a number of properly matched features and a fraction of properly resolved swapped feature pairs. Our tool matched practically all drifted features (96 %) and the vast majority of swapped feature pairs (91 %). We compared our results with two open-source feature matching algorithms: OpenMS, and LWBMatch. OpenMS had high feature matching precision, it matched the majority of drifted features (80 %). However, its accuracy drastically decreased when analyzing only swapped feature pairs (61 %). LWBMatch had a significantly lower matching precision, it matched 24 % of drifted features and only 3 % of swapped feature pairs.

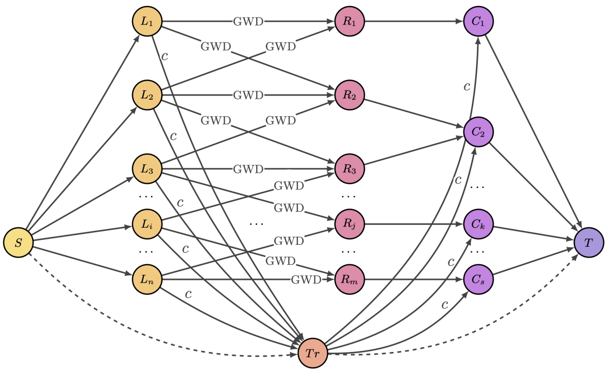

Figure 4. Flow network for finding the optimal feature matching. This matching is computed between selected chromatogram, denoted by n features $L_1, \ldots, L_n$ and m features from the rest of chromatograms, denoted by nodes $R_1, \ldots, R_m$. Clusters are denoted by s nodes $C_1, \ldots, C_s$. Nonzero costs are described by edge labels. The cost between features L_i and features R_j is equal to GWD between them. Additional node Tr ('trash') gives the possibility to not match the feature with cost c. Every edge has capacity equal to 1, except edge between S (source) and Tr and edge between Tr and T (sink) with capacities equal to $\max\{0, s - n\}$ and $\max\{0, n - s\}$ respectively (at most one of them has nonzero capacity). Edges between $R_1, \ldots, R_m$ and $C_1, \ldots, C_s$ give the restriction that aby feature can be matched with at most one cluster. As a result, we take all matchings (L_i, C_k). We recognize the consensus feature by its cluster.

Algorithm validation on benchmark datasets

We evaluated the accuracy of our method by comparing alignment quality on public benchmark datasets. We reproduced the evaluation protocol from Lange et al. study [36] (further referred to as the CAAP study). We analyzed two proteomic datasets from CAAP evaluation: P1 and P2, and one metabolomic: M1. P1 set contained the analysis of *E. coli* protein extracts and consisted of 6 fractions at different salt bumps, every fraction in 2 different runs. Analogously, P2 contained the analysis of protein extract from *M. smegmatis* in 5 fractions every in 3 replicated runs. M1 contained the analysis of leaf tissue extract from *A. thaliana* in 44 repeated runs. To assess the correctness of alignment algorithms, the authors of the CAAP study proposed alignment precision and alignment recall measures (cf. Methods). Moreover, as proposed by the authors of SIMA algorithm [18], we computed the F-score, which is a harmonic mean of alignment precision and recall.

We analyzed sets P1, P2, and M1 and compared Alignstein with the results of the OpenMS alignment algorithm [15] from the CAAP study. We chose OpenMS because it achieved significantly better results than the other tools and represented a state-of-the-art solution at the time of the original study. Moreover, we included in comparison the available results of algorithms published more recently: MZMine 2 [17], SIMA [18], MassUntagler [22] (only P1 set), and Wandy et al. [24]. We measured the time of alignment computation, results are presented in Table 2.

Alignstein obtained highly competitive results in CAAP evaluation. For the P1 dataset, it matched perfectly almost all features, its precision and recall were on average 0.94, similarly to MZmine 2 and OpenMS (cf. Table 1, Supplementary Table S1). SIMA obtained slightly worse results and the rest of the tools obtained lower values than SIMA. Interestingly, all tools achieved average alignment precision and recall no higher than 0.94. It may suggest that improperly matched features either are too distant to be matched based on LC-MS information or ground-truth is misspecified.

For the P2 set, we achieved the highest average alignment recall (on average 0.82), i.e. our approach had a minimal num-

Table 1. Comparison of alignment precision (P), alignment recall (R), and F-score (F). For P1 and P2 sets, average over fractions is computed. Dash marks result not presented in the original papers.

		Alignstein	OpenMS	MZMine 2	Wandy et al.	SIMA	MassUntangler
P1	P	0.94	0.94	0.94	0.88	0.94	0.87
	R	0.94	0.94	0.94	0.89	0.92	0.79
	F	0.94	0.94	0.94	0.88	0.93	0.83
P2	P	0.74	0.83	0.68	0.72	0.72	-
	R	0.83	0.72	0.75	0.72	0.75	-
	F	0.78	0.77	0.71	0.72	0.74	-
M1	P	0.88	0.69	0.74	-	0.75	-
	R	0.91	0.87	0.91	-	0.92	-
	F	0.89	0.77	0.82	-	0.83	-

ber of unmatched features (cf. Table 1, Supplementary Table S2). It had a lower precision on average equal to 0.73 and was second only to OpenMS. Overall, we obtained the best average F-score value, equal to 0.77.

For the M1 dataset, Alignstein achieved competitive results: precision equal to 0.88, recall 0.91, F-score 0.89. This confirms that Alignstein's scales effectively with the number of input chromatograms.

Application to the detection of specific biomarkers

Alignstein can detect specific biomarkers in medical applications or biological analysis. To verify this, we analyzed the dataset from Barranger et al.'s work [3]. It contained LC-MS/MS chromatograms of intestinal protein from marine mussels exposed in vivo to various benzo[a]pyrene (BaP) concentrations (0, 5, 50, 100 $\mu g/L$).

We checked if Alignstein recognizes tandem mass spectra (MS/MS) information by spatial properties of LC-MS features. To assess this, we detected LC-MS features and annotated them with peptide MS/MS identifications. The accuracy of alignment was quantified using proposed identification recall (IR) defined as follows. We chose all repeating identifications that have annotated features and computed a fraction of them that were properly aligned (cf. Methods). For every BaP concentration, we computed IR for all aligned technical replicates of the sample. We achieved satisfactory results IR equal to 81 %, 78 %, 85 %, 86 % respectively for BaP concentrations 0, 5, 50, and 100 $\mu g/L$. As a baseline, we repeated this analysis for the OpenMS algorithm, which achieved similar results with IR equal to 81 %, 76 %, 85 %, and 83 %. Moreover, we calculated the IR separately for every subset of all aligned chromatograms (see Methods). This demonstrated that our approach uniformly treats all chromatograms (cf. Fig. 5a and Supplementary Fig. S1).

Moreover, we checked if Alignstein can detect corresponding biomarkers for LC-MS measurements of samples under different experimental conditions. For this purpose, we repeated the analysis above by aligning chromatograms across all BaP concentrations. The overall IR was equal to 85 %. Contrary to the previous experiment, IR for OpenMS has fallen to 0.75 %. Alignstein's results were analogously as earlier uniform over all chromatogram subsets (cf. Fig. 5b) with IR values not lower than 67 %, reaching even 100 % for some subsets of repeated identifications. This proves that, despite the varying experimental conditions, our solution is able to correctly align the vast majority of corresponding features without accuracy loss. Finally, this experiment shows that it may be applied as a tool for biomarkers screening in LC-MS analysis.

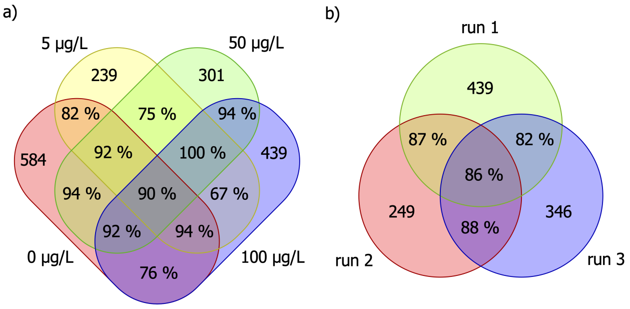

Figure 5. Identification recall calculated separately for every chromatogram subset. a) For aligned chromatograms over all BaP concentrations. b) For replicates (1, 2, 3) of the sample of 100 $\mu g/L$ BaP concentration. Sets represent chromatograms, intersections contain identification recall, and the non-overlapping part of the sets contains the number of feature-annotated identifications.

Discussion

Alignstein is a novel, original algorithm for LC-MS alignment based on the GWD feature dissimilarity measure. This allows for incorporating not only distances between features, but also their spatial differences and thus more accurate feature alignment. The GWD emerges to be a key solution for correctly aligning signals with a swapped elution order, as demonstrated in the previous sections.

In addition to correctly resolving feature swaps, Alignstein has more advantages over the majority of alignment algorithms. It requires no prior feature identification, so LC-MS data without additional tandem mass spectra suffice as input to the algorithm. Moreover, our approach makes no assumptions about the characteristics of the analyzed chromatograms, so it is not limited to one type of data (e.g. proteomic or metabolomic). Still, specific properties of the analyzed data (e.g. maximum drift size) can be passed as algorithm parameters. Finally, it treats uniformly all analyzed chromatograms, and thus it does not require a reference sample.

Alignstein requires only the prior feature detection as a data preprocessing step. Although approaches with this requirement are criticized [8, 20], we argue that the analysis with detected features is more accurate than the analysis of raw chromatograms. Properly executed feature detection effectively discriminates regions of high signal-to-noise ratio from chro-

Table 2. Alignstein runtimes on benchmark CAAP datasets.

P1	P2	M1
10 sec	15 sec	15 min 38 sec

For the P1 and P2 dataset wall-time was measured for a single fraction. For the M1 dataset wall-time is measured for the whole dataset.

matograms [37]. Moreover, multidimensional feature detection is crucial for collecting information about coeluting ions (e.g. isotopic envelopes of compounds). Without this, any alignment algorithm might yield inaccurate results by aligning signals across isotopic envelopes.

Besides advantages, Alignstein has also limitations. It correctly matches the vast majority of features, but it happens to fail to match distant features. This mismatch can be explained by interpreting GWD as a sum of two costs: the cost of transporting the feature along the RT (to eliminate drift) and the cost of transformation (to incorporate feature-feature spatial differences). For a pair of distant, corresponding features, the cost of transport along the RT far exceeds the cost of transformation. For this reason, even highly dissimilar but much closer features may camouflage the correct feature correspondence. This can be particularly troublesome for complex datasets having a significant number of features, which are densely packed within chromatograms. This limitation can be only partially corrected by adjusting GWD parameters because the majority of corresponding feature pairs have RT differences of less than 10 seconds (cf. Supplementary Fig. S2) and thus the GWD parameters must be optimized for small feature distances. One of the possible solutions is to incorporate additional information for alignment, for example, MS/MS data. Thus, we plan to extend our algorithm to deal with LC-MS/MS datasets in a data-independent acquisition mode.

In conclusion, Alignstein correctly aligns chromatograms as we have shown in the biomarkers detection experiment, by reproducing the CAAP evaluation study, as well as in swaps resolving computational comparison. Its highly competitive matching accuracy is the result of applying the Generalized Wasserstein Distance as a feature dissimilarity measure, which allows matching features without reducing feature spatial information or the dimension of data. Thus, Alignstein is capable of detecting non-obvious signal patterns and finding optimal alignment. Our solution provides a solid basis for further applications of optimal transport theory to the multidimensional problems of automated analysis in mass spectrometry. We hope that the optimal transport-based distances will become a new paradigm as a measure of spectra dissimilarity and will allow the construction of highly effective, robust, and accurate algorithms for mass spectrometry analysis.

Methods

Feature dissimilarity measure

The most common approach to comparing mass spectra is a cosine-like similarity score [28, 38]. Despite its popularity, this class of scores is not applicable to feature alignment, because they are not scalable with dimension and cannot effectively compare spectra of significantly different molecules [29]. To address these limitations, we propose the Wasserstein distance [39] with additional generalizations [14, 40] as a feature dissimilarity measure.

The Wasserstein distance is a metric based on optimal transport theory. It describes how to optimally transform one feature into the other one. These transformations may include shifting the signal as well as splitting or combining the signal between peaks (cf. Fig. 2). Formally, suppose that we have two discrete features μ and ν so that $\mu(x)$ is the intensity of μ at M/Z value x. Then we define the transport plan T so that $T(x,y)$ corresponds to the amount of signal that is transported from a peak x of feature μ to peak y of feature ν. The transport cost is the sum of amounts of transport between all pairs of peaks multiplied by the distance between peaks:

$$\sum_{x,y} T(x,y) \cdot d(x,y), \tag{1}$$

where $d(x,y)$ is a distance between peaks x and y. For this setup, we have chosen $d(x,y)$ to be ℓ_1 distance (a Manhattan distance). The Wasserstein distance W is the minimal transport cost of all possible transport plans T:

$$W(\mu,\nu) = \min_T \sum_{x,y} T(x,y) \cdot d(x,y). \tag{2}$$

Besides effectiveness, we observed that Wasserstein distance unsatisfactorily deals with noisy features. To overcome this limitation, we use a generalization of Wasserstein distance (GWD) as proposed by Chizat et al. [40]. GWD differs mainly from Wasserstein distance by the possibility to omit transporting part of the signal with a constant penalty. More specifically, GWD allows omitting the transport of signal on a distance larger than the user-defined λ parameter with a constant penalty proportional to λ and the amount of not transported signal:

$$W(\mu,\nu) = \min_T \sum_{x,y} \Big(T(x,y) \cdot d(x,y) + \lambda \cdot F(T_\mu, \mu) + \lambda \cdot F(T_\nu, \nu) \Big), \tag{3}$$

where T_μ and T_ν are the marginals of the transport plan. F is a divergence chosen so that the approximation of the transport plan T to features μ and ν is possible. To compute GWD, we regularize it with the entropic term which allows for fast and numerically stable computation, using a scaling Sinkhorn-Knopp approximation algorithm [41]. Fully formal distance derivation is available in Supplementary Material sections 1-2.

Alignstein algorithm scheme

Alignstein is an algorithm for LC-MS alignment. Here, the alignment is formulated as finding the correspondence of detected features, which represent the same chemical entities (e.g. ions, compounds). Specifically, the algorithm takes chromatograms with detected features as an input, and the outcome of the algorithm is a list of consensus features. Consensus features are sets of corresponding features from distinct chromatograms. The algorithm outline is depicted in Fig. 1 and pseudocode is available in Algorithm 1.

Alignstein starts with feature preprocessing. If the features are not provided by the user, it detects them using the Feature Finder algorithm from the OpenMS package. Features are represented as a set of all signal peaks contained within the boundaries of detected features. For further processing, Alignstein normalizes features and scales the RT so that the RT dimension variation becomes of a similar order of magnitude as the M/Z dimension variation. Scaling is done by dividing the RT by a factor proportional to the ratio of the average feature length (along the RT axis) and the average feature width (along the M/Z axis).

After preprocessing, alignment consists of two phases: the centroid clustering phase and then the feature matching phase. During the former one, centroids of features from all chromatograms are clustered using Mini-Batch K-Means [42] and hierarchical clustering algorithms. Clustering is computed to create candidates for consensus features, which are then verified by the feature matching phase. During this phase, the algorithm searches for pairs of the most similar features across all chromatograms. It is done by finding the feature matching of minimal cost, where the cost is equal to the sum of GWDs

between the matched features. We interpret this problem as finding the maximal flow of minimum cost in an appropriately designed flow network, in which we compare iteratively every chromatogram with the rest of the chromatograms. In Fig. 4, we show a flow network for a single chromatogram. The flow of minimum cost is obtained using the network simplex [43] algorithm. This minimization problem has formulated restrictions, such that features may be not matched with a constant penalty, or at most one feature may be matched to features within one cluster. These restrictions assure that features are matched appropriately as required, e.g. the algorithm treats uniformly all input chromatograms, at most one feature from every chromatogram would be chosen to consensus features, etc. Finally, consensus features are created via clusters that have been matched as most similar in optimal matching. A more detailed algorithm description is available in Supplementary Material section 3.

In the special case, when only two chromatograms are aligned, the clustering phase is omitted and consensus features are created by finding the optimal matching between two feature sets (cf. Supplementary Fig. S3).

Implementation details

Alignstein is implemented as a Python 3 package and available at [44]. It uses C++ implementation of GWD in the MassSinkhornmetry package available at [45]. For centroid clustering, we used clustering algorithms implemented in the scikit-learn package [46, 47]. For solving the minimum cost flow problem, we used the data structures and algorithms implemented in NetworkX [48, 49] package.

Alignstein benchmarking details

We validated the Alignstein algorithm by reproducing the evaluation protocol from the Critical Assessment of Alignment Procedures (CAAP) study [36]. It was the analysis and comparison of 7 alignment algorithms: OpenMS [15], msInspect [50], MZmine 1 [51], SpecArray [52], XAlign [53], and XCMS [54].

We analyzed two proteomic datasets (P1 and P2) and one metabolomic (M1) from the CAAP study. For all sample sets, preparation, and analysis protocols are described in the original study. For metabolomic set as well as for every fraction at different salt bumps (0, 20, 40, 60, 80, and 100 mM ammonium chloride) of both proteomic sets, the authors prepared a set of ground-truth consensus features, which represent feature correspondence over chromatograms of significantly high confidence.

To assess the accuracy of alignment, the authors of the CAAP study proposed the generalization of precision and recall as alignment precision and alignment recall. Alignment precision measures how the given ground-truth consensus feature was split over tool consensus features, i.e. it reflects the number of false positives. Alignment recall measures how many features of a given ground-truth consensus feature are found by the algorithm, i.e. it reflects the number of false negatives. Both alignment precision and recall are calculated as an arithmetic mean over all ground-truth consensus features. Furthermore, the authors of SIMA [18] and Wandy et al. [24] proposed the F-score which is the harmonic mean of alignment precision and alignment recall ($\frac{2 \cdot P \cdot R}{P+R}$, where P is alignment precision and R is alignment recall) to express the balance of alignment precision and alignment recall.

We used input chromatograms as mzML and mzXML files and features as featureXML files provided by authors of the CAAP study. We measured alignment precision and recall using an evaluation script written in R programming language by the authors of this study. Computation was done on a computer with Linux operating system and 24 Intel Xeon E5-2620 2.10 GHz processors + 62 GB RAM. We measured wall time using Linux built-in `time` command. More details on CAAP benchmarking are provided in the Supplementary Material section 4.

Algorithm 1: Alignstein algorithm

Input: chromatograms $ch_1, \ldots, ch_n$,
Result: consensus features $c_1, \ldots, c_s$
// Preprocessing phase
$features_1, \ldots, features_n \leftarrow$
  preprocessFeatures($ch_1, \ldots, ch_n$)
// Centroid clustering phase
$centroids \leftarrow \emptyset$
forall $features_i$ **do**
  $centroids \leftarrow$
   $centroids \cup$ {centroids of all features from $features_i$}
$clusters \leftarrow$ cluster $centroids$
// Matching phase
forall $features_i$ **do**
  $matching_i \leftarrow$ match features from $features_i$ to
   $\bigcup_{j \in \{1,\ldots,n\} \setminus i} features_j$
$c_1 \ldots, c_m \leftarrow$
  createConsensusFromMatchings($matching_1, \ldots, matching_n$)

Mussels toxicological response experiment summary

For assessment of Alignstein's ability to detect specific biomarkers, we analyzed chromatograms originally created in the work of Barranger et al. [3]. The original study aimed to measure the effects of polluting the marine mussels (*Mytilus galloprovincialis*) environment with fullerene (C60) and benzo[a]pyrene (BaP). For this purpose, the authors performed a proteomic analysis.

Mussels were collected in Trebar with Strand, Cornwall, UK, and were exposed in vivo to C60 and BaP at concentrations 0, 5, 50, and 100 $\mu g/L$ as described in the original study. For proteomic analysis, mussel intestinal proteins were collected. After digestion and purification, the peptides were analyzed by the LC-MS/MS system with data-dependent acquisition (DDA) mode as described in Sequiera et al. [55]. In summary, peptides were separated on Dionex Ultimate 3000 RSLC nanoflow system: Acclaim PepMap C18 nano column 75 $\mu m \times 25$ cm, 3 μm, 100 Å plus bypass, a linear gradient of 96 % buffer A (0.5 % Acetic Acid) and 4 % buffer B (80 % acetonitrile in 0.5 % acetic acid) to 60 % buffer A and 40 % buffer B, the flow rate of 300 ml/min for 120 minutes. Separated analytes were analyzed in an Orbitrap Velos Pro FTMS (Thermo Finnigan) with positive ion mode ionization with Proxeon nanospray ESI source. In each run, the 10 most abundant ions were further analyzed with additional collision-induced dissociation (CID) fragmentation (30 % collision energy) in a linear ion trap spectrometer. For every BaP concentration from 0, 5, 50, to 100 $\mu g/L$ three replicates were obtained. Collected chromatograms for all BaP exposure levels were deposited in the ProteomeXchange Consortium PRIDE repository (PXD013805) [56, 57].

Data analysis for detection of repeating biomarkers

In downloaded chromatograms, we identified peptides using Comet [58, 59]. We obtained the database for peptide identification from the original work (taxa Mollusca, subcategory

Bivalvia from Uniprot KnowledgeBase, and contaminants from Global Proteome Machine [60]). The most important Comet search parameters were: peptide mass tolerance of 10 ppm, trypsin as search enzyme, concatenated decoy search, and allowed missed enzyme cleavages no higher than 2.

We detected features in chromatograms using the OpenMS algorithm Feature Finder in the Centroided version. We annotated the detected LC-MS features with MS/MS Comet identifications. Peptide MS/MS identifications were represented in LC-MS by retention time in seconds and the ratio of the precursor neutral mass to the assumed charge. The feature was annotated with identification when LC-MS representation of identification was enclosed within feature boundaries. For further analysis, we considered annotated features.

For calculating IR, we computed the number of repeating identifications over chromatograms. For every repeating identification, we checked if annotated features were properly matched by Alignstein. IR was calculated as a ratio of the number of correctly aligned annotated repeating identifications and the total number of annotated repeating identifications.

Number of swaps estimation

We analyzed two replicates of 0 $\mu g/L$ BaP concentration in the dataset described in the previous section. Computation was done for all pairs of annotated features with repeating identification in both chromatograms. We computed the fraction of these pairs that were swapped, i.e. a feature pair was considered as a swap when the computed feature RT means of the same identifications in two replicates were in a different order.

Availability of source code and requirements

- Project name: Alignstein,
- Project home page: [44],
- Operating systems: Linux, macOS,
- Programming language: Python 3,
- Other requirements: Python 3.6 or higher; dependency packages: MassSinkhornmetry, pyOpenMS, NumPy, SciPy, NetworkX, scikit-learn,
- License: MIT,
- Any restrictions to use by non-academics: none,
- RRID: SCR_022483,
- bio.tools ID: alignstein.

Availability of supporting data and materials

The Marine Mussels dataset was obtained from ProteomeXchange Consortium PRIDE repository under accession no. PXD013805. Benchmark datasets (P1, P2, M1), as well as evaluation script, were obtained from the CAAP webpage at [61]. Datasets P1 and P2 are originally available in Open Proteomic Database [62]. Snapshots of Alignstein source code and other data further supporting this work are openly available in the GigaScience repository, GigaDB [63].

Declarations

List of abbreviations

BaP: benzo[a]pyrene; CAAP: Critical Assessment of Alignment Procedures; C60: fullerene; CID: collision-induced dissociation. DDA: data-dependent acquisition; GWD: generalized Wasserstein distance; HPLC: high-performance liquid chromatography; IR: identification recall; LC-MS: liquid chromatography mass spectrometry; MS/MS: tandem mass spectrometry; M/Z: mass-to-charge ratio; RT: retention time; UPLC: ultra-performance liquid chromatography;

Consent for publication

Not applicable.

Competing Interests

The authors declare that they have no competing interests.

Funding

GS was supported by Polish National Science Center grant number 2019/33/N/ST6/02949. AG and BM were supported by Polish National Science Center grant number 2018/29/B/ST6/00681.

Author's Contributions

GS implemented and verified the algorithm. AG conceived the idea of the project and discussed the results. BM designed the algorithm and supervised the work. GS, AG, and BM co-wrote the manuscript.

Acknowledgments

The authors thank Marta Nowak for her comments on the manuscript.

References

1. Runkle KB, Kharbanda A, Stypulkowski E, Cao XJ, Wang W, Garcia BA, et al. Inhibition of DHHC20-Mediated EGFR Palmitoylation Creates a Dependence on EGFR Signaling. Molecular Cell 2016 May;62(3):385–396.
2. Sethi MK, Thaysen-Andersen M, Kim H, Park CK, Baker MS, Packer NH, et al. Quantitative proteomic analysis of paired colorectal cancer and non-tumorigenic tissues reveals signature proteins and perturbed pathways involved in CRC progression and metastasis. Journal of Proteomics 2015;126:54–67.
3. Barranger A, Langan LM, Sharma V, Rance GA, Aminot Y, Weston NJ, et al. Antagonistic Interactions between Benzo[a]pyrene and Fullerene (C60) in Toxicological Response of Marine Mussels. Nanomaterials 2019 Jul;9(7):987.
4. Tomechko SE, Liu G, Tao M, Schlatzer D, Powell CT, Gupta S, et al. Tissue Specific Dysregulated Protein Subnetworks in Type 2 Diabetic Bladder Urothelium and Detrusor Muscle. Molecular & Cellular Proteomics 2015 Mar;14(3):635–645.
5. Zhou B, Xiao JF, Tuli L, Ressom HW. LC-MS-based metabolomics. Mol BioSyst 2012;8(2):470–481.
6. Snyder LR, Kirkland JJ, Dolan JW. Introduction to Modern Liquid Chromatography. John Wiley & Sons, Inc.; 2009.
7. K Magnus Åberg and Erik Alm and Ralf J O Torgrip. The correspondence problem for metabonomics datasets. Analytical and Bioanalytical Chemistry 2009 feb;394(1):151–162.
8. Smith R, Ventura D, Prince JT. LC-MS alignment in theory and practice: a comprehensive algorithmic review. Briefings in Bioinformatics 2013 Nov;16(1):104–117.

9. Xianyin Lai and Lianshui Wang and Frank A Witzmann. Issues and Applications in Label-Free Quantitative Mass Spectrometry. International Journal of Proteomics 2013 jan;2013:1–13.
10. Lindemann, Claudia and Thomanek, Nikolas and Hundt, Franziska and Lerari, Thilo and Meyer, Helmut E and Wolters, Dirk and Marcus, Katrin. Strategies in relative and absolute quantitative mass spectrometry based proteomics. Biological chemistry 2017;398(5-6):687–699.
11. Jürgen Cox and Marco Y Hein and Christian A Luber and Igor Paron and Nagarjuna Nagaraj and Matthias Mann. Accurate Proteome-wide Label-free Quantification by Delayed Normalization and Maximal Peptide Ratio Extraction, Termed MaxLFQ. Molecular & Cellular Proteomics 2014 Sep;13(9):2513–2526.
12. James A Dowell and Logan J Wright and Eric A Armstrong and John M Denu. Benchmarking Quantitative Performance in Label-Free Proteomics. ACS Omega 2021 jan;6(4):2494–2504.
13. Li, Yunong and Li, Liang. Retention time shift analysis and correction in chemical isotope labeling liquid chromatography/mass spectrometry for metabolome analysis. Rapid Communications in Mass Spectrometry 2020;34(S1):e8643.
14. Peyré G, Cuturi M. Computational Optimal Transport: With Applications to Data Science. Foundations and Trends® in Machine Learning 2019;11(5-6):355–607.
15. Lange E, Gröpl C, Schulz-Trieglaff O, Leinenbach A, Huber C, Reinert K. A geometric approach for the alignment of liquid chromatography—mass spectrometry data. Bioinformatics 2007 07;23(13):i273–i281.
16. Lommen A. MetAlign: Interface-Driven, Versatile Metabolomics Tool for Hyphenated Full-Scan Mass Spectrometry Data Preprocessing. Analytical Chemistry 2009 Mar;81(8):3079–3086.
17. Pluskal T, Castillo S, Villar-Briones A, Orešič M. MZmine 2: Modular framework for processing, visualizing, and analyzing mass spectrometry-based molecular profile data. BMC Bioinformatics 2010 Jul;11(1).
18. Voss B, Hanselmann M, Renard BY, Lindner MS, Köthe U, Kirchner M, et al. SIMA: Simultaneous Multiple Alignment of LC/MS Peak Lists. Bioinformatics 2011 Feb;27(7):987–993.
19. Zhang Z. Retention Time Alignment of LC/MS Data by a Divide-and-Conquer Algorithm. Journal of the American Society for Mass Spectrometry 2012 Feb;23(4):764–772.
20. Gupta S, Ahadi S, Zhou W, Röst H. DIAlignR Provides Precise Retention Time Alignment Across Distant Runs in DIA and Targeted Proteomics. Molecular & Cellular Proteomics 2019 Apr;18(4):806–817.
21. Wu CT, Wang Y, Wang Y, Ebbels T, Karaman I, Graça G, et al. Targeted realignment of LC-MS profiles by neighbor-wise compound-specific graphical time warping with misalignment detection. Bioinformatics 2020 Jan;36(9):2862–2871.
22. Ballardini R, Benevento M, Arrigoni G, Pattini L, Roda A. MassUntangler: A novel alignment tool for label-free liquid chromatography–mass spectrometry proteomic data. Journal of Chromatography A 2011 Dec;1218(49):8859–8868.
23. Wang J, Lam H. Graph-based peak alignment algorithms for multiple liquid chromatography-mass spectrometry datasets. Bioinformatics 2013 Jul;29(19):2469–2476.
24. Wandy J, Daly R, Breitling R, Rogers S. Incorporating peak grouping information for alignment of multiple liquid chromatography-mass spectrometry datasets. Bioinformatics 2015 Feb;31(12):1999–2006.
25. Tsugawa H, Cajka T, Kind T, Ma Y, Higgins B, Ikeda K, et al. MS-DIAL: data-independent MS/MS deconvolution for comprehensive metabolome analysis. Nature Methods 2015 May;12(6):523–526.
26. The, Matthew and Käll, Lukas. Focus on the spectra that matter by clustering of quantification data in shotgun proteomics. Nature communications 2020;11(1):1–12.
27. Moorthy AS, Kearsley AJ. 4. In: Pattern Similarity Measures Applied to Mass Spectra Springer International Publishing; 2020. p. 43–53.
28. Kim S, Zhang X. Comparative Analysis of Mass Spectral Similarity Measures on Peak Alignment for Comprehensive Two-Dimensional Gas Chromatography Mass Spectrometry. Computational and Mathematical Methods in Medicine 2013;2013:1–12.
29. Huber F, Ridder L, Verhoeven S, Spaaks JH, Diblen F, Rogers S, et al. Spec2Vec: Improved mass spectral similarity scoring through learning of structural relationships. PLOS Computational Biology 2021 Feb;17(2):e1008724.
30. Lacki MK, Valkenborg D, Startek MP. IsoSpec2: Ultrafast Fine Structure Calculator. Analytical Chemistry 2020 Jun;92(14):9472–9475.
31. Levitsky LI, Klein JA, Ivanov MV, Gorshkov MV. Pyteomics 4.0: Five Years of Development of a Python Proteomics Framework. Journal of Proteome Research 2018 Dec;18(2):709–714.
32. Ciach MA, Miasojedow B, Skoraczyński G, Majewski S, Startek M, Valkenborg D, et al. Masserstein: Linear regression of mass spectra by optimal transport. Rapid Communications in Mass Spectrometry 2021 Jan;.
33. Permiakova, Olga and Guibert, Romain and Kraut, Alexandra and Fortin, Thomas and Hesse, Anne-Marie and Burger, Thomas. CHICKN: extraction of peptide chromatographic elution profiles from large scale mass spectrometry data by means of Wasserstein compressive hierarchical cluster analysis. BMC bioinformatics 2021;22(1):1–30.
34. Seifert NA, Prozument K, Davis MJ. Computational optimal transport for molecular spectra: The fully discrete case. The Journal of Chemical Physics 2021 Nov;155(18):184101.
35. Seifert NA, Prozument K, Davis MJ. Computational optimal transport for molecular spectra: The semi-discrete case. The Journal of Chemical Physics 2022 Apr;156(13):134117.
36. Lange E, Tautenhahn R, Neumann S, Gröpl C. Critical assessment of alignment procedures for LC-MS proteomics and metabolomics measurements. BMC Bioinformatics 2008 Sep;9(1).
37. Zohora FT, Rahman MZ, Tran NH, Xin L, Shan B, Li M. DeepIso: A Deep Learning Model for Peptide Feature Detection from LC-MS map. Scientific Reports 2019 Nov;9(1).
38. Frank AM, Monroe ME, Shah AR, Carver JJ, Bandeira N, Moore RJ, et al. Spectral archives: extending spectral libraries to analyze both identified and unidentified spectra. Nature methods 2011;8(7):587–591.
39. Kantorovich LV. Mathematical Methods of Organizing and Planning Production. Management Science 1960 Jul;6(4):366–422.
40. Chizat L, Peyré G, Schmitzer B, Vialard FX. Scaling algorithms for unbalanced optimal transport problems. Mathematics of Computation 2018 feb;87(314):2563–2609.
41. Knopp P, Sinkhorn R. Concerning nonnegative matrices and doubly stochastic matrices. Pacific Journal of Mathematics 1967;21(2):343 – 348.
42. Sculley D. Web-scale k-means clustering. In: Rappa M, Jones P, Freire J, Chakrabarti S, editors. Proceedings of the 19th international conference on World wide web - WWW '10 New York, NY, USA: ACM Press; 2010. p. 1177–1178.
43. Király Z, Kovács P. Efficient implementations of minimum-cost flow algorithms. Acta Univ Sapientiae, Inform 2012;4(1):67–118.

44. Alignstein; Version: 1.0. https://github.com/grzsko/Alignstein.
45. MassSinkhornmetry;. https://github.com/grzsko/MassSinkhornmetry, accessed: 8 June 2022.
46. Pedregosa F, Varoquaux G, Gramfort A, Michel V, Thirion B, Grisel O, et al. Scikit-learn: Machine Learning in Python. Journal of Machine Learning Research 2011;12:2825–2830.
47. scikit-learn; Version: 1.1.1. https://scikit-learn.org/stable/index.html.
48. Hagberg AA, Schult DA, Swart PJ. Exploring Network Structure, Dynamics, and Function using NetworkX. In: Varoquaux G, Vaught T, Millman J, editors. Proceedings of the 7th Python in Science Conference Pasadena, CA USA; 2008. p. 11 – 15.
49. NetworkX; Version: 2.8.3. https://networkx.org/.
50. Bellew M, Coram M, Fitzgibbon M, Igra M, Randolph T, Wang P, et al. A suite of algorithms for the comprehensive analysis of complex protein mixtures using high-resolution LC-MS. Bioinformatics 2006;22(15):1902–1909.
51. Katajamaa M, Orešič M. Processing methods for differential analysis of LC/MS profile data. BMC bioinformatics 2005;6(1):1–12.
52. Li Xj, Eugene CY, Kemp CJ, Zhang H, Aebersold R. A Software Suite for the Generation and Comparison of Peptide Arrays from Sets of Data Collected by Liquid Chromatography-Mass Spectrometry* S. Molecular & Cellular Proteomics 2005;4(9):1328–1340.
53. Zhang X, Asara JM, Adamec J, Ouzzani M, Elmagarmid AK. Data pre-processing in liquid chromatography–mass spectrometry-based proteomics. Bioinformatics 2005;21(21):4054–4059.
54. Smith CA, Want EJ, O'Maille G, Abagyan R, Siuzdak G. XCMS: Processing Mass Spectrometry Data for Metabolite Profiling Using Nonlinear Peak Alignment, Matching, and Identification. Analytical Chemistry 2006 Jan;78(3):779–787.
55. Sequiera GL, Sareen N, Sharma V, Surendran A, Abu-El-Rub E, Ravandi A, et al. High throughput screening reveals no significant changes in protein synthesis, processing, and degradation machinery during passaging of mesenchymal stem cells. Canadian Journal of Physiology and Pharmacology 2019 Jun;97(6):536–543.
56. Vizcaíno JA, Deutsch EW, Wang R, Csordas A, Reisinger F, Ríos D, et al. ProteomeXchange provides globally coordinated proteomics data submission and dissemination. Nature Biotechnology 2014 Mar;32(3):223–226.
57. Perez-Riverol Y, Csordas A, Bai J, Bernal-Llinares M, Hewapathirana S, Kundu DJ, et al. The PRIDE database and related tools and resources in 2019: improving support for quantification data. Nucleic Acids Research 2018 Nov;47(D1):D442–D450.
58. Eng JK, Jahan TA, Hoopmann MR. Comet: An open-source MS/MS sequence database search tool. PROTEOMICS 2012 Dec;13(1):22–24.
59. Eng JK, Hoopmann MR, Jahan TA, Egertson JD, Noble WS, MacCoss MJ. A Deeper Look into Comet—Implementation and Features. Journal of the American Society for Mass Spectrometry 2015 Jun;26(11):1865–1874.
60. The Global Proteome Machine;. https://www.thegpm.org/, accessed: 28 September 2022.
61. Critical Assessment of Alignment Procedures Data and evaluation scripts;. https://msbi.ipb-halle.de/msbi/caap, accessed: 28 September 2022.
62. Open Proteomics Database;. http://data.marcottelab.org/MSdata/OPD/, accessed: 28 September 2022.
63. Skoraczyński G, Gambin A, Miasojedow B, Supporting data for "Alignstein: optimal transport for improved LC-MS retention time alignment"; 2022. http://dx.doi.org/10.5524/102267. GigaScience Database.

Click here to access/download
Supplementary Material
suppl_material.pdf
