# Supplementary material for: Alignstein: Optimal transport for improved LC-MS retention time alignment
Source: Gigascience. 2022 Nov 3;11:giac101. doi: 10.1093/gigascience/giac101 (PMC9633278; doi:10.1093/gigascience/giac101)
Supplement: giac101_Supplemental_File [file giac101_supplemental_file.pdf]

Supplementary material

to article *Alignstein: optimal transport for improved LC-MS retention time alignment*

Grzegorz Skoraczyński Anna Gambin Błażej Miasojedow

1 The Wasserstein distance definition

Recently, the Wasserstein distance has emerged as a practical metric for comparing probability measures. Also, it transfers naturally to mass spectrometry. Mass spectrum represents the distribution of charged ions in a spectrometer detector, and thus it corresponds to some probability measure. In the present work, we generalize the spectrum to a two-dimensional chromatogram subset, a feature. Unless otherwise stated, the definitions for a spectrum and feature are the same. In the present text, we introduce a fully formal definition of the Wasserstein distance and provide a handy notation for generalizing the Wasserstein distance in Section 2.

Suppose, that we have a spectrum (feature) represented by a probability measure μ. In the present work, we evaluate centroided spectra (features), so we may assume that the measure μ is discrete and finite. We assume also that all the spectrum's (feature's) peak intensities sum up to 1, i.e. it is normalized (all intensities are divided) by its total ion current. Further in the text, we denote spectrum (feature) and measure μ interchangeably. For given measure μ, we denote a support $\mathrm{Supp}(\mu) = (x_1, \dots, x_n)^\intercal$, which corresponds to M/Z values and measure values $\boldsymbol{\mu} = (\mu_1, \dots, \mu_n)^\intercal$ corresponding to intensities located in $\mathrm{Supp}(\mu)$. Further in the text, for notation simplicity, we assume that i iterates over $1, \dots, n$ and j iterates over $1, \dots, m$.

Suppose we have two measures (spectra, features) μ with $\mathrm{Supp}(\mu) = (x_1, \dots, x_n)^\intercal$ and values $\boldsymbol{\mu} = (\mu_1, \dots, \mu_n)^\intercal$ and ν with $\mathrm{Supp}(\nu) = (y_1, \dots, y_m)^\intercal$ and values $\boldsymbol{\nu} = (\nu_1, \dots, \nu_m)^\intercal$. For optimal transport formulation, we define cost matrix $\boldsymbol{M} \in \mathbb{R}^{n,m}_{\geqslant 0}$, so that

$$M_{i,j} = d(x_i, y_i),$$

where $d(x_i, y_i)$ is a distance between x_i and y_j. Cost matrix $\boldsymbol{M}$ describes the cost of transportation of the unit of ion current from peak located in M/Z x_i of the spectrum (feature) μ to peak located in M/Z y_i of the spectrum (feature) ν. Here, we use the ℓ^1 metric as a cost. For one dimensional spectra this is a distance between two M/Z-s, i.e. $d(i, j) = |x_i - y_j|$. For 2-dimensional features, it is a sum of distances in M/Z and linearly scaled RT.

Define also set U as a set of all couplings between measures μ and ν as

$$U(\mu, \nu) = \left\{ \boldsymbol{T} \in \mathbb{R}^{n,m}_{\geqslant 0} \;\middle|\; \boldsymbol{T} \cdot \mathbb{1}_m = \boldsymbol{\mu}, \boldsymbol{T}^\intercal \cdot \mathbb{1}_n = \boldsymbol{\nu} \right\}.$$

We call the coupling $\boldsymbol{T} \in \mathbb{R}^{n,m}_{\geqslant 0}$ as a transport plan, where $T_{i,j}$ describes amount of ion current transported from x_i of spectrum (feature) μ to y_i of spectrum (feature) ν. For given transport plan $\boldsymbol{T}$ and cost matrix $\boldsymbol{M}$ we define a transport cost as the sum of multiplied transported amounts of ion current and costs, i.e.

$$\sum_{i,j} T_{ij} M_{ij}$$

The Wasserstein distance is an optimal transport cost, i.e. a solution to the following minimization problem

$$OT_{\boldsymbol{M}}(\mu, \nu) = \min_{\boldsymbol{T} \in U(\mu,\nu)} \sum_{i,j} T_{ij} M_{ij}. \tag{1}$$

If $\boldsymbol{T^*}$ is the transport plan of minimal cost (optimal transport plan), then the Wasserstein distance is

$$d^W(\mu, \nu) = \sum_{i,j} T^*_{ij} M_{ij}.$$

The problem of finding optimal $\boldsymbol{T}^*$ is a linear programming problem, but for one-dimensional spectra, it can be computed in linear time [4].

Example. Suppose that we want to compute the optimal transport between two spectra depicted in the figure on the right. The spectrum μ can be defined so that

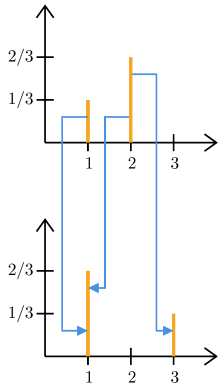

$$\boldsymbol{\mu} = \left(\frac{1}{3}, \frac{2}{3}\right)^{\mathsf{T}} \qquad \mathrm{Supp}(\mu) = (1,2)^{\mathsf{T}}$$

and spectrum ν can be defined so that

$$\boldsymbol{\nu} = \left(\frac{2}{3}, \frac{1}{3}\right)^{\mathsf{T}} \qquad \mathrm{Supp}(\nu) = (1,3)^{\mathsf{T}}.$$

Cost matrix is

$$\boldsymbol{M} = \begin{bmatrix} 0 & 2 \\ 1 & 1 \end{bmatrix}.$$

Then the optimal transport plan is (depicted with blue lines):

$$\boldsymbol{T} = \begin{bmatrix} \frac{1}{3} & 0 \\ \frac{1}{3} & \frac{1}{3} \end{bmatrix}.$$

The cost of optimal transport (the Wasserstein distance) equals:

$$d^W(\mu, \nu) = \frac{1}{3} \cdot 0 + 0 \cdot 2 + \frac{1}{3} \cdot 1 + \frac{1}{3} \cdot 1 = \frac{2}{3}.$$

2 Generalized Wasserstein distance derivation

2.1 Entropic penalizing

The minimization problem (1) is a linear programming problem and can be solved in polynomial time using the simplex algorithm [5]. We propose, however, using the Sinkhorn-Knopp scaling approximation algorithm [8] for speeding up computation and better numerical stability. For a broader introduction to Wasserstein distance computation, consult the Cuturi's book [9].

We start with reformulating the minimization problem (1) by adding the entropic penalty:

$$OT^{\varepsilon}_{\boldsymbol{M}}(\mu,\nu) = \min_{\boldsymbol{T}\in U(\mu,\nu)} \sum_{i,j} T_{ij} M_{ij} - \varepsilon h(\boldsymbol{T}) \tag{2}$$

where $h(\boldsymbol{T})$ is entropic regularization term:

$$h(\boldsymbol{T}) = -\sum_{i,j} T_{ij} \log T_{ij}.$$

Analogously, if $\boldsymbol{T}^*$ is the transport plan that minimizes $OT^{\varepsilon}_{\boldsymbol{M}}(\mu,\nu)$, i.e. is the optimal transport plan, then the cost of optimal transport is

$$d^{\varepsilon}(\mu,\nu) = \sum_{i,j} T^*_{ij} M_{ij}.$$

Adding entropic penalty, makes the objective function strongly convex and not only speeds up computation time but also assures better numerical stability.

To compute the solution of problem (2), we notice that the optimal solution is unique [12] and has the form

$$(\boldsymbol{T}^*)_{ij} = u_i v_j e^{-M_{ij}/\varepsilon}$$

for two scaling variables $\boldsymbol{u} \in \mathbb{R}^n$ and $\boldsymbol{v} \in \mathbb{R}^m$. The algorithm for computing the optimal transport will have the scaling scheme (Sinkhorn-Knopp algorithm [8]) as depicted below.

Algorithm 1: The scheme of the Sinkhorn algorithm.

Data: $\boldsymbol{M}, \varepsilon, \boldsymbol{\mu}, \boldsymbol{\nu}$
Result: optimal $\boldsymbol{T}$
$\boldsymbol{T} \leftarrow e^{-\boldsymbol{M}/\varepsilon}$;
while *not converged* **do**
  scale $\boldsymbol{T}$ rows such that row sums match to $\boldsymbol{\mu}$;
  scale $\boldsymbol{T}$ columns such that column sums match to $\boldsymbol{\nu}$;
return $\boldsymbol{T}$

For more precise algorithm formulation, we notice that we can conveniently rewrite $\boldsymbol{T}$ as

$$\boldsymbol{T} = \operatorname{diag}(\boldsymbol{u}) \boldsymbol{K} \operatorname{diag}(\boldsymbol{v})$$

for a matrix $\boldsymbol{K}$. We define for $\boldsymbol{x} \in \mathbb{R}^n$, the $\operatorname{diag}(\boldsymbol{x})$ as $n \times n$ matrix with diagonal $\boldsymbol{x}$ and zero otherwise. This implies that

$$\operatorname{diag}(\boldsymbol{u}) \boldsymbol{K} \operatorname{diag}(\boldsymbol{v}) \cdot \mathbb{1}_m = \boldsymbol{u} \odot (\boldsymbol{K}\boldsymbol{v}) = \boldsymbol{\mu}$$

and

$$\operatorname{diag}(\boldsymbol{v}) \boldsymbol{K}^{\top} \operatorname{diag}(\boldsymbol{u}) \cdot \mathbb{1}_n = \boldsymbol{v} \odot (\boldsymbol{K}^{\top}\boldsymbol{u}) = \boldsymbol{\nu}.$$

So the scaling iterations will be as below:

$$\boldsymbol{u}^{(l+1)} \leftarrow \frac{\boldsymbol{\mu}}{\boldsymbol{K}\boldsymbol{v}^{(l)}}$$
$$\boldsymbol{v}^{(l+1)} \leftarrow \frac{\boldsymbol{\nu}}{\boldsymbol{K}^\top \boldsymbol{u}^{(l+1)}}$$

and the algorithm gets the form as below.

Algorithm 2: The scheme of the Sinkhorn algorithm.

Data: $\boldsymbol{M}, \varepsilon, \boldsymbol{\mu}, \boldsymbol{\nu}$
Result: optimal $\boldsymbol{T}$
$\boldsymbol{K} \leftarrow e^{-\boldsymbol{M}/\varepsilon}$;
$\boldsymbol{v} \leftarrow \mathbb{1}_m$;
while *not converged* **do**
  $\boldsymbol{u} \leftarrow \frac{\boldsymbol{\mu}}{\boldsymbol{K}\boldsymbol{v}}$;
  $\boldsymbol{v} \leftarrow \frac{\boldsymbol{\nu}}{\boldsymbol{K}^\top \boldsymbol{u}}$;
return $(\boldsymbol{u}_i \cdot K_{ij} \cdot \boldsymbol{v}_j)_{i,j}$

The algorithm above is the τ-approximate solution of the unregularized OT problem (1) in $O(n^2 \log(n)\tau^{-3})$ operations [1].

2.2 Dealing with noise

We observed that the Wasserstein distance does not deal well with noise, trying to match it with signal and vice-versa. To cope with this problem, we implemented the Generalized Wasserstein Distance for unbalanced measures as proposed by Chizat [3]. For this purpose, we generalize our problem

$$OT^{\varepsilon,F}_{\boldsymbol{M}}(\mu,\nu) = \min_{\boldsymbol{T}\in U(\mu,\nu)} \sum_{i,j} T_{ij}M_{ij} - \varepsilon h(\boldsymbol{T}) + F(\boldsymbol{T}\cdot \mathbb{1}_m|\boldsymbol{\mu}) + F(\boldsymbol{T}^\top \cdot \mathbb{1}_n|\boldsymbol{\nu}),$$

where distance is

$$d^\varepsilon_F(\mu,\nu) = \sum_{i,j} T^*_{ij}M_{ij} + F(\boldsymbol{T}^*\cdot \mathbb{1}_m|\boldsymbol{\mu}) + F(\boldsymbol{T}^{*\top} \cdot \mathbb{1}_n|\boldsymbol{\nu}).$$

F is a divergence that allows us to ‘approximate the optimal solution to μ and ν measures. From a practical point of view, this allows us to ignore the noise and penalize not transporting noise with constant penalty. There are several divergences that can be used, but choose the total variation diverge, which performed best for our application:

$$F(\boldsymbol{x}|\boldsymbol{y}) = \lambda\,\mathrm{TV}(\boldsymbol{x}|\boldsymbol{y}) = ||\boldsymbol{x}-\boldsymbol{y}||_{\mathrm{TV}}$$

which for our setup is

$$||\boldsymbol{a}||_{\mathrm{TV}} = \sum_i |a_i|.$$

For this problem, scaling steps of the algorithm can be generalized using the proximal operator:

$$\mathrm{prox}^{\mathrm{KL}}_{F/\varepsilon}(\boldsymbol{z}) = \operatorname*{argmin}_{\boldsymbol{s}\in\mathbb{R}^n} F(\boldsymbol{s}) + \varepsilon\,\mathrm{KL}(\boldsymbol{s}|\boldsymbol{z}),$$

where $KL(\boldsymbol{x}|\boldsymbol{y})$ is Kullback-Leibler divergence:

$$\mathrm{KL}(\boldsymbol{x}|\boldsymbol{y}) = \sum_i x_i \log\left(\frac{x_i}{y_i}\right).$$

For total variation divergence, the proximal operator has the formula:

$$\operatorname{prox}^{\mathrm{KL}}_{F/\varepsilon}(\boldsymbol{s}) = \min\left\{\boldsymbol{s}\cdot e^{\frac{\lambda}{\varepsilon}}, \max\{\boldsymbol{s}\cdot e^{-\frac{\lambda}{\varepsilon}}, \boldsymbol{p}\}\right\}.$$

Now, scaling steps have the form as below:

$$\boldsymbol{u} \leftarrow \frac{\operatorname{prox}^{\mathrm{KL}}_{F/\varepsilon}(\boldsymbol{K}\boldsymbol{v})}{\boldsymbol{K}\boldsymbol{v}}$$

$$\boldsymbol{v} \leftarrow \frac{\operatorname{prox}^{\mathrm{KL}}_{F/\varepsilon}(\boldsymbol{K}^{\top}\boldsymbol{u})}{\boldsymbol{K}^{\top}\boldsymbol{u}}$$

and finally, the algorithm for finding optimal transport has the form as below.

Algorithm 3: The scheme of the Sinkhorn algorithm.

Data: $\boldsymbol{M}, \lambda, \varepsilon, \boldsymbol{\mu}, \boldsymbol{\nu}$
Result: optimal $\boldsymbol{T}$
$\boldsymbol{K} \leftarrow e^{-\lambda \boldsymbol{M}}$;
$\boldsymbol{v} \leftarrow \mathbb{1}_m$;
while *not converged* **do**
  $\boldsymbol{u} \leftarrow \frac{\operatorname{prox}^{\mathrm{KL}}_{F/\varepsilon}(\boldsymbol{K}\boldsymbol{v})}{\boldsymbol{K}\boldsymbol{v}}$;
  $\boldsymbol{v} \leftarrow \frac{\operatorname{prox}^{\mathrm{KL}}_{F/\varepsilon}(\boldsymbol{K}^{\top}\boldsymbol{u})}{\boldsymbol{K}^{\top}\boldsymbol{u}}$;
return $(\boldsymbol{u}_i \cdot K_{ij} \cdot \boldsymbol{v}_j)_{i,j}$

3 Alignstein algorithm details

3.1 Algorithm formulation

Alignstein is an algorithm for LC-MS alignment by feature matching, i.e. for chromatograms with detected features, it finds the correspondence of features. As an input, it takes chromatograms to be aligned and the result is a list of consensus features, i.e. a set of corresponding features from distinct chromatograms. The Alignstein algorithm pseudocode is shown in the main text.

Preprocessing phase In the preprocessFreatures function, features are collected and prepared for further analysis as summarized in function preprocessFeatures. Features can be provided by the user. Otherwise, Alignstein detects them using the FeatureFinderCentroided algorithm from OpenMS package [10] on the fly. Usually, software-detected features are represented only by their boundaries (e.g. RT and M/Z spans or convex hulls) and thus Alignstein collects all signal peaks contained inside feature boundaries before the run. In the beginning, features are denoised and normalized. For further processing, Alignstein scales RT so that the RT axis variation is roughly at the same level of magnitude as the M/Z axis variation. Scaling is done by dividing R/T by a factor proportional to $\frac{AL_{RT}}{AW_{M/Z}}$, where AL_{RT} is the average feature length (along the RT axis) and AW_{MZ} is average feature width (along the M/Z axis).

Function preprocessFeatures

```
Input: chromatograms ch_1, ..., ch_n,
Result: feature sets features_1, ..., features_n
forall ch_i do
    features_det_i ← parse or detect features in chromatogram ch_i
    features_i ← ∅
    forall feature_det ∈ features_det_i do
        feature ← collected signal from ch_i represented by feature_det
        feature ← normalize feature
        push feature to features_i
AL_RT ← average feature length from features_1, ..., features_n
AW_M/Z ← average feature width from features_1, ..., features_n
forall features_i do
    forall feature ∈ features_i do
        scale RT of feature by factor proportional to AL_RT / AW_M/Z
```

Centroid clustering phase After preprocessing, Alignstein starts with the centroid clustering phase which consists of collecting centroids of all features and clustering them. The aim of centroid clustering is to create candidates for consensus features, which are further verified during the matching phase. Because the number of centroids from all chromatograms may be significantly large, centroid clustering is done in two steps: firstly, we split the whole space into several smaller pieces using the mini-batch k-means algorithm [11], then we do the final clustering using hierarchical clustering.

Matching phase During the matching phase, Alignstein searches for feature similarities over chromatograms. It is done by matching features from every chromatogram towards features of the rest of the chromatograms. Formally, for every chromatogram i, its set of features, $features_i$ is matched to the union of features from the rest of the chromatograms: $REST_i =$

$\bigcup_{j \in \{1,\ldots,n\} \setminus i} features_j$. Matching can be expressed as the problem of finding the optimal matching between features from $features_i$ to the rest of features $REST_i$, so that:

- every feature from $features_i$ can be matched with at most one feature from $REST_i$,
- every feature from $REST_i$ can be matched by at most one feature from $features_i$,
- for every cluster c_k, at most one feature $f_j \in REST_i$ contained within c_k can be matched,
- for every cluster c_k, either one feature contained within it can be matched or no feature is matched with a constant penalty,
- cost of matching two features f_1, f_2 is proportional to GWD between f_1 and f_2,
- feature from $feature_i$ can be not matched with a constant penalty,
- cost of matching is a sum of costs of matched features and penalties for not matching,
- result matching is a matching of minimal cost.

The constant penalty of not matching allows omitting to match of too different features, i.e. those with GWD larger by a penalty threshold. Moreover, there is a restriction that for every cluster only one feature can be matched. This assures that consensus features contain at most one feature from every chromatogram. Further, every cluster consensus feature is created as described in Consensus features creation.

We reduce the above minimization problem to finding the maximal flow of minimum cost in a network shown in Fig. 4. Alignstein finds the optimal solution using the primal network simplex algorithm [7] implemented in Networkx package [6].

For efficiency reasons, we compute GWD only for pairs of features that are in the same region of interest, i.e. are close enough to be matched. It allows omitting computing GWD between obviously too distant features, e.g. features with an M/Z distance larger than 100 Da. Checking if features are in the same region of interest is done by computing feature centroids' distance.

Consensus features creation The consensus feature for cluster c_k is obtained as a union of features matched to any feature contained in c_k as shown in Function createConsensusFeatures

Function createConsensusFeatures

Input: matchings $matching_1, \ldots, matching_n$,
// *$matching_i$ describes matching of features from i-th chromatogram to clusters of features from the rest of chromatograms*
Result: consensus features $c_1, \ldots, c_s$
$s \leftarrow$ number of clusters
$c_1, \ldots, c_s \leftarrow \varnothing, \ldots, \varnothing$
forall $matching_i$ **do**
    forall $\langle$matched $feature$, cluster $c_k\rangle \in matching_i$ **do**
        $c_k \leftarrow c_k \cup \{feature\}$

3.2 The special case of two chromatograms

In the special case, when only two chromatograms are aligned, the clustering phase is omitted and features are matched directly. Optimal matching is computed by minimizing the global cost of matching, i.e. the sum of GWDs between matched features and penalties for not matching.

Every feature can be either matched with exactly one feature from another chromatogram or not matched with a constant penalty. Here, the constant penalty for not matching can be interpreted as a maximal distance up to which features are considered similar. Analogously as in general algorithm formulation, we reduce the feature matching problem to finding the maximum flow of minimal cost in the network described in Supplementary Figure 3.

4 Algorithm benchmarking additional notes

4.1 Dataset M1

M1 dataset lacks the spatial information of analyzed features. For this reason, we reproduced feature detection using XCMS3 with parameters detailed in the CAAP study (`method = "centWave", peakwidth = c(20, 50), snthresh = 5, ppm = 12`). We matched features from the study with newly generated features by checking if the previous feature representation falls within the bounding box of new features. Such matched features were further input of the evaluation script.

4.2 Data preparation additional notes

Except for the P1, P2, and M1 sets, the CAAP study consisted also of the analysis of another metabolomic dataset M2. However, due to the currently limited data availability, we omitted this dataset. Originally, in the CAAP study, only a fraction of all detected features were aligned. The evaluation protocol lacks, however, a detailed description of initial feature filtering for further alignment. Thus, we decided to filter features to those existing in ground-truth.

4.3 Additional comments to CAAP benchmarking results

OpenMS alignment algorithm performed best in the CAAP study. Originally, the authors of this study evaluated the OpenMS version 1.0. Its alignment algorithm was reimplemented in 2012 and the previous version is no longer bundled with the OpenMS package. We reproduced the evaluation of the CAAP study on the current version of OpenMS. Unfortunately, the current alignment algorithm is achieving significantly worse results despite strenuous attempts to adjust the algorithm parameters to the data. Its alignment precision and recall are on average 60 percentage points lower than the results reported in the CAAP study.

The majority of alignment algorithms are not compared with any tool [13]. This results in difficulties in broad comparing Alignstein with the majority of algorithms. Thus, there is a constant need for dedicated LC-MS alignment assessment of currently state-of-the-art alignment software that not only complements CAAP with other datasets but also selects the best currently available alignment algorithm. To the best of the authors' knowledge, CAAP is the only assessment of LC-MS alignment algorithms done on real datasets and thus it is widely used for validation. The limited availability of benchmark datasets may result in a growing tendency to analyze algorithms only on data from CAAP work and, therefore, to overfit to this dataset. The presented results verify that Alignstein is not affected by this problem. Not only it was validated on multiple datasets, but also almost all results are not significantly outstanding than other best-performing tools. One exception is an outstanding average recall for the P2 dataset, but it is consistent with the algorithm's design so that it maximizes the number of matches up to the user-defined parameter of the cost threshold.

References

[1] Jason Altschuler, Jonathan Niles-Weed, and Philippe Rigollet. Near-linear time approximation algorithms for optimal transport via sinkhorn iteration. In I. Guyon, U. Von Luxburg, S. Bengio, H. Wallach, R. Fergus, S. Vishwanathan, and R. Garnett, editors, *Advances in Neural Information Processing Systems*, volume 30. Curran Associates, Inc., 2017.

[2] Audrey Barranger, Laura M. Langan, Vikram Sharma, Graham A. Rance, Yann Aminot, Nicola J. Weston, Farida Akcha, Michael N. Moore, Volker M. Arlt, Andrei N. Khlobystov, James W. Readman, and Awadhesh N. Jha. Antagonistic interactions between benzo[a]pyrene and fullerene (c60) in toxicological response of marine mussels. *Nanomaterials*, 9(7):987, July 2019.

[3] Lénaïc Chizat, Gabriel Peyré, Bernhard Schmitzer, and François-Xavier Vialard. Scaling algorithms for unbalanced optimal transport problems. *Mathematics of Computation*, 87(314):2563–2609, feb 2018.

[4] Michał Aleksander Ciach, Błażej Miasojedow, Grzegorz Skoraczyński, Szymon Majewski, Michał Startek, Dirk Valkenborg, and Anna Gambin. Masserstein: Linear regression of mass spectra by optimal transport. *Rapid Communications in Mass Spectrometry*, January 2021.

[5] George B. Dantzig. Origins of the simplex method, June 1990.

[6] Aric A. Hagberg, Daniel A. Schult, and Pieter J. Swart. Exploring network structure, dynamics, and function using networkx. In Gaël Varoquaux, Travis Vaught, and Jarrod Millman, editors, *Proceedings of the 7th Python in Science Conference*, pages 11 – 15, Pasadena, CA USA, 2008.

[7] Zoltán Király and Péter Kovács. Efficient implementations of minimum-cost flow algorithms. *Acta Univ. Sapientiae, Inform.*, 4(1):67–118, 2012.

[8] Paul Knopp and Richard Sinkhorn. Concerning nonnegative matrices and doubly stochastic matrices. *Pacific Journal of Mathematics*, 21(2):343 – 348, 1967.

[9] Gabriel Peyré and Marco Cuturi. Computational optimal transport: With applications to data science. *Foundations and Trends® in Machine Learning*, 11(5-6):355–607, 2019.

[10] Hannes L Röst, Timo Sachsenberg, Stephan Aiche, Chris Bielow, Hendrik Weisser, Fabian Aicheler, Sandro Andreotti, Hans-Christian Ehrlich, Petra Gutenbrunner, Erhan Kenar, Xiao Liang, Sven Nahnsen, Lars Nilse, Julianus Pfeuffer, George Rosenberger, Marc Rurik, Uwe Schmitt, Johannes Veit, Mathias Walzer, David Wojnar, Witold E Wolski, Oliver Schilling, Jyoti S Choudhary, Lars Malmström, Ruedi Aebersold, Knut Reinert, and Oliver Kohlbacher. OpenMS: a flexible open-source software platform for mass spectrometry data analysis. *Nature Methods*, 13(9):741–748, aug 2016.

[11] D. Sculley. Web-scale k-means clustering. In *Proceedings of the 19th International Conference on World Wide Web*, WWW '10, page 1177–1178, New York, NY, USA, 2010. Association for Computing Machinery.

[12] Richard Sinkhorn. A relationship between arbitrary positive matrices and doubly stochastic matrices. *The Annals of Mathematical Statistics*, 35(2):876–879, jun 1964.

[13] R. Smith, D. Ventura, and J. T. Prince. Novel algorithms and the benefits of comparative validation. *Bioinformatics*, 29(12):1583–1585, April 2013.

Supplementary figures

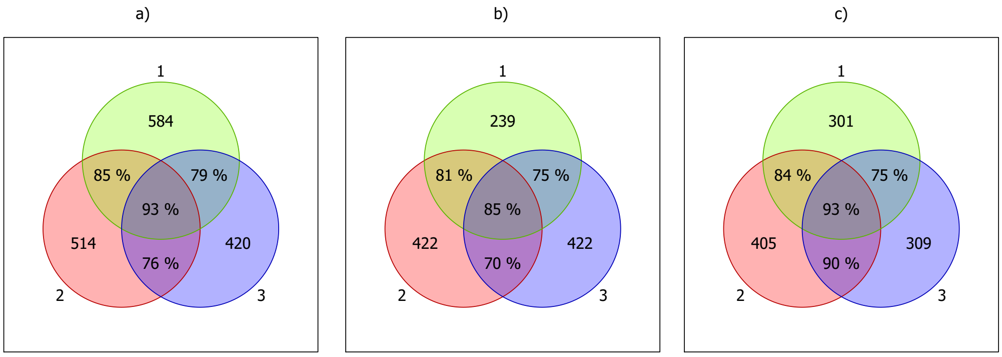

Supplementary Figure S 1: Identification recall calculated separately for identifications repeating in every chromatogram subsets. a) For replicates of the sample with $0\mu g/L$ BaP. b) For replicates of the sample with $5\mu g/L$ BaP. c) For replicates of the sample with $50\mu g/L$ BaP. Sets represent replicates (chromatograms) of the same experiments, the inconjunct part of the set contains the number of feature-annotated identifications, and conjunctions contain identification recall.

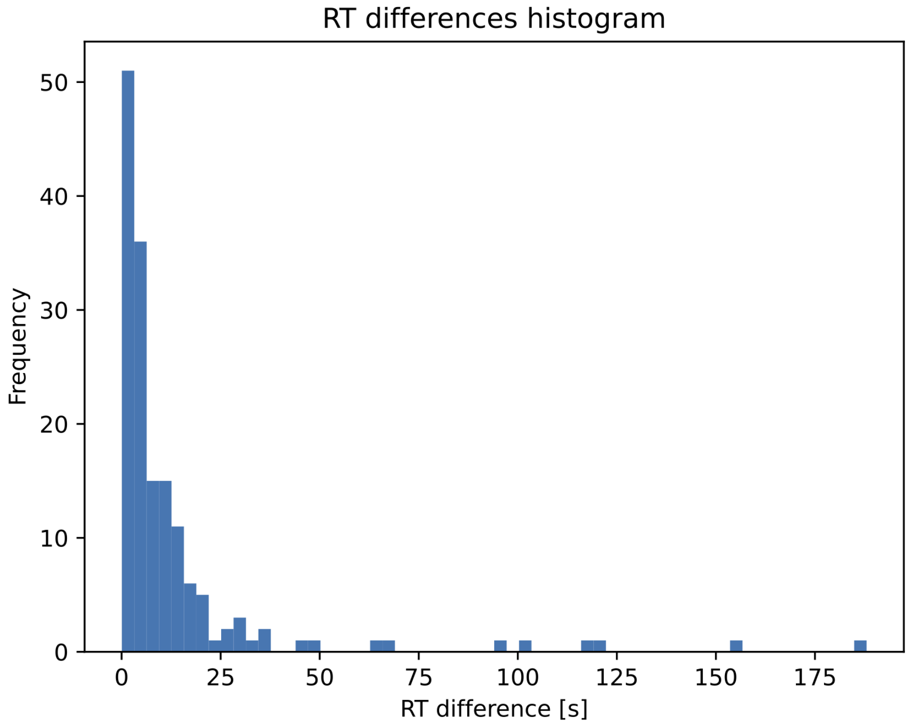

Supplementary Figure S 2: Histogram of RT centroid differences between feature pairs annotated with the same identification. The histogram is computed for chromatograms from Barranger et al. work [2], replicates of a sample with $0 \mu g/L$ BaP. For better readability, outliers over 200 seconds are omitted. The majority of RT differences are not greater than 10 seconds.

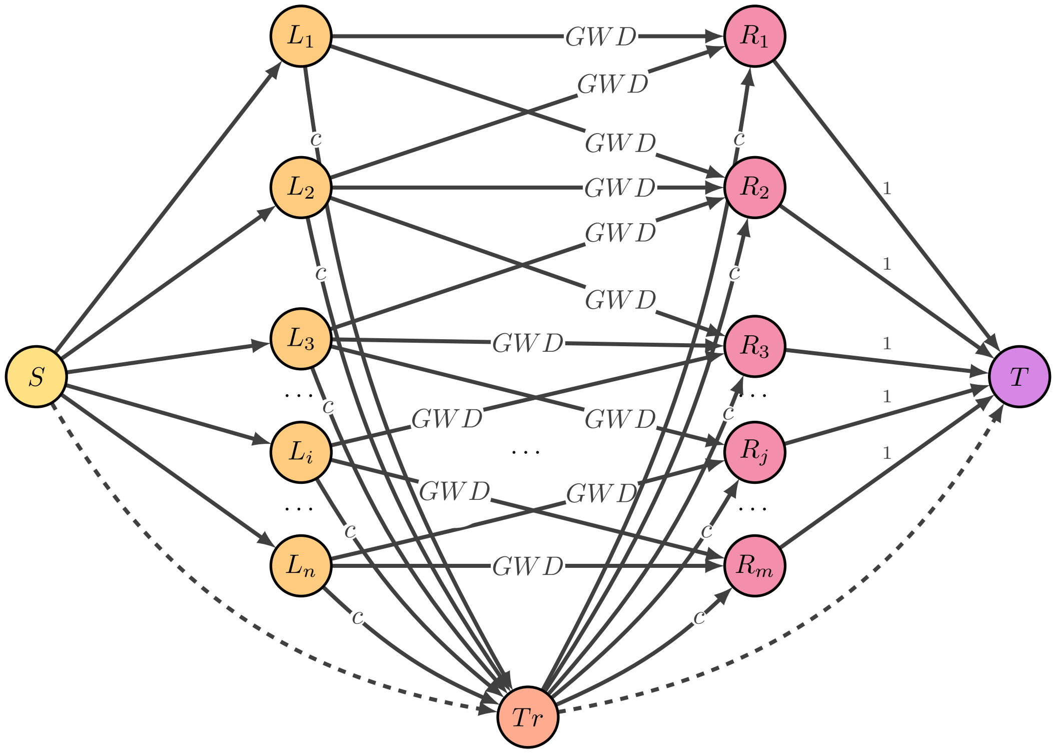

Supplementary Figure S 3: Flow network for finding the optimal feature matching between n features of one chromatogram denoted by nodes $L_1, \ldots, L_n$ and m features from the other chromatogram, denoted by nodes $R_1, \ldots, R_m$. Nonzero costs are described by edge labels. The cost between features L_i and features R_j is equal to GWD between them. Additional node Tr ('trash') gives the possibility to not match the feature with cost c. Every edge has capacity equal to 1, except edge between S (source) and Tr and edge between Tr and T (sink) with capacities equal to $\max\{0, s-n\}$ and $\max\{0, n-s\}$ respectively (at most one of them has nonzero capacity). As a result, we take all matchings (L_i, R_j).

Supplementary tables

Subset		Alignstein	OpenMS	MZMine 2	Wandy et al.	SIMA	MassUntangler
0-20	P	0,94	0,86	0,86	0,75	0,86	0,87
	R	0,94	0,86	0,86	0,79	0,83	0,76
	F	0,94	0,86	0,86	0,77	0,84	0,81
20-40	P	0,9	0,93	0,93	0,95	0,97	0,86
	R	0,9	0,93	0,93	0,95	0,94	0,73
	F	0,90	0,93	0,93	0,95	0,95	0,79
40-60	P	0,92	0,93	0,94	0,89	0,94	0,87
	R	0,92	0,93	0,94	0,86	0,91	0,8
	F	0,92	0,93	0,94	0,87	0,92	0,83
60-80	P	0,94	0,96	0,97	0,86	0,94	0,8
	R	0,94	0,96	0,97	0,9	0,92	0,68
	F	0,94	0,96	0,97	0,88	0,93	0,74
80-100	P	0,98	0,97	0,97	0,92	0,98	0,93
	R	0,98	0,97	0,97	0,92	0,96	0,89
	F	0,98	0,97	0,97	0,92	0,97	0,91
100-120	P	0,94	0,96	0,96	0,9	0,96	0,89
	R	0,94	0,96	0,96	0,92	0,95	0,87
	F	0,94	0,96	0,96	0,91	0,95	0,88

Supplementary Table S 1: Detailed results for P1 set in CAAP comparison. P stands for alignment precision, R stands for alignment recall, and F stands for F-score.

Subset		Alignstein	OpenMS	MZMine 2	Wandy et al.	SIMA
0	P	0,72	0,77	0,49	0,49	0,55
	R	0,84	0,65	0,56	0,48	0,61
	F	0,77	0,70	0,52	0,48	0,58
20	P	0,67	0,92	0,78	0,79	0,75
	R	0,79	0,76	0,93	0,81	0,89
	F	0,72	0,83	0,85	0,80	0,81
40	P	0,82	0,76	0,77	0,78	0,81
	R	0,88	0,74	0,78	0,82	0,75
	F	0,85	0,75	0,77	0,80	0,78
80	P	0,78	0,80	0,61	0,68	0,74
	R	0,86	0,70	0,61	0,66	0,63
	F	0,82	0,75	0,61	0,67	0,68
100	P	0,70	0,90	0,75	0,85	0,77
	R	0,80	0,75	0,88	0,85	0,89
	F	0,74	0,82	0,81	0,85	0,83

Supplementary Table S 2: Detailed results for P2 set in CAAP comparison. P stands for alignment precision, R stands for alignment recall, and F stands for F-score.
