# Supplementary material for: Alignstein: Optimal transport for improved LC-MS retention time alignment
Source: Gigascience. 2022 Nov 3;11:giac101. doi: 10.1093/gigascience/giac101 (PMC9633278; doi:10.1093/gigascience/giac101)
Supplement: giac101_Response_to_Reviewer_Comments_Original_Submission [file giac101_response_to_reviewer_comments_original_submission.pdf]

Author's Response To Reviewer Comments

Close

Dear Editor,

We enclose a revised manuscript of Alignstein's algorithm. We appreciate all reviewers' comment, because they helped to improve the quality of the manuscript. We have modified our manuscript accordingly. Please find below the responses to the comments of all reviewers.

Best regards
Grzegorz Skoraczyński, Anna Gambin, Błażej Miasojedow

Responses to reviewers' comments:

Reviewer 1

> The manuscript presents a new algorithm for retention time alignment in LC-MS
> data. The algorithm called alignstein uses a more appropriate multi-dimensional
> alignment strategy than existing algorithms. The authors should however
> investigate more of the available methods that try to achieve a similar goal
> (e.g., quandenser). In addition to this the introduction should be rewritten a
> bit to make it comprehensible. In the results the authors did not convince me
> of the better performance of alignstein. I admit that this might be due to the
> available data or benchmarks, but if this is the case I would have at least
> expected a discussion about these limitations. I suggest a major revision as I
> think the authors are likely able to address all my concerns.
>
> Major
>
> 1. Why is this needed? And what statistical analysis are you talking about? Do
> you mean quantification? In my opinion that is not a statistical analysis. "RT
> drift needs to be corrected prior to statistical LC-MS analysis."

We are grateful for this remark and we rewrote the whole paragraph as follows:

'RT drift requires a correction, usually named the RT alignment. It results in the correspondence of signals across runs. For example, in proteomics, the signal correspondence of the same peptides is needed for further applying label-free quantification (LFQ) for which samples must be measured separately. Moreover, for LFQ techniques, we cannot obtain the correspondence any other way because analytes do not have any additional information, such as metabolic labels, or chemical tags.'

> 2. The authors mention "Typically, it is caused by external conditions of
> variable nature, such as temperature [7].", but before it was mentioned it is
> hard to control experimental variables to counter retention time drift.
> External temperature is not hard to control. You can have chamber heating,
> pre-column heating, and even post-column heating.

Indeed, too simplistic example. We extended a list of experimental conditions affecting LC separation and rewrote the entire paragraph as below addressing also the other remarks (Minor 3, Minor 4):

'RT drift can be corrected by the experimental protocol only to a limited

extent. It may change the whole gradient or affect only single peaks. These changes may be caused by various reasons such as the unstable mobile phase, the column change or degradation, sample chemical instability, or imprecise experiment setup.'

> 3. I think the authors should have a look at "Quandenser" as it resolves a lot
> of the issues raised. The, M., Käll, L. Focus on the spectra that matter by
> clustering of quantification data in shotgun proteomics. Nat Commun 11, 3234
> (2020). https://doi.org/10.1038/s41467-020-17037-3

We did not know about Quandenser. Thanks for mentioning it. It looks really good, we find this idea of MS2 clustering very brilliant. However, it still reduces features into single points incorporating only the information about: minimal RT, maximal RT, average RT, single M/Z, charge, and total intensity. When we were designing Alingstein, we aimed to take advantage of incorporating all features' signal information (span over RT and isotopic envelope) by its shape and the distribution of all signals.

We mentioned Quandenser in a paragraph listing other feature-matching algorithms. We address the same issues to Quandenser as raised in the original manuscript, i.e.

"To the best of the authors' knowledge, all matching algorithms reduce multidimensional features to one-dimensional extracted ion chromatograms or a single points with monoisotopic peak M/Z and average RT value, ignoring the information of isotopic envelope or feature span over the RT dimension. Without feature spatial characteristics and information of coeluting ions, elution order swaps are practically undetectable."

> 4. I would recommend the authors to look more into the literature between
> quantification and retention time alignment. The manuscript in its current form
> does not speak of quantification. While most people apply retention time
> alignment to better quantification. Also, the authors would have good
> benchmarks to test the performance of their algorithm through the use of these
> controlled experiments. E.g., see: Dowell, J. A., Wright, L. J., Armstrong, E.
> A., & Denu, J. M. (2021). Benchmarking quantitative performance in label-free
> proteomics. ACS omega, 6(4), 2494-2504.

Quantification is an important task. However, Alignstein is not designed as a quantification tool. The goal of Aligstein is to make the best possible RT alignment before quantification. Extending it to quantification benchmarking is out of the scope of the current paper.

Moreover, the proposed work benchmarks quantification techniques but it does not document RT alignment. Thus, we cannot derive any ground truth from this work and it does not allow for accurate RT benchmarking.

We described alignment as a preprocessing step of quantification as detailed in answer to Major 1.

> 5. I am very skeptical of evaluating performance on swapped signal with
> simulated data. I would urge the authors to seek further validation that does
> not involve simulated data. Or at least explain in detail why this is not
> possible to perform on experimentally acquired data.

The reason for using simulated data are limits of the other benchmarking techniques: limited ground-truth coverage of input datasets. Usually, we cannot correctly identify even half of our input data. Thus, we cannot check the accuracy of the alignment of features not covered by the ground truth. We checked the general accuracy of alignment on several real datasets: P1, P2, Mussels, and additionally, after revision, M1. Simulated data gives us the full information about the true correspondence. We used it only for benchmarking

swaps detection, which affects only a fraction of features and thus is prone to ground-truth incompleteness.

> 6. Differences between Alignstein and OpenMS seem relatively small, if they
> perform equally is there another reason to use alignstein? Why is this
> difference so small when alignstein uses a much more sophisticated (according
> to the authors) algorithm for retention time alignment? Is the benchmark not
> suitable for evaluating?

While we were designing Alignstein, we wanted to fulfill several design goals: e.g. 1. no assumption about monotonicity of RT drift, 2. no need for reference sample, i.e. aligning fully uniformly with regard to the input chromatograms, 3. no need for any prior data identification.

The most challenging is goal 2, which is not met by OpenMS and practically almost all other algorithms. OpenMS takes either a reference sample or arbitrarily chooses a chromatogram with the greatest number of features and aligns all other datasets to this one. This may partially skew the results.

Moreover, since the CAAP study, OpenMS has changed its alignment implementation, remaining the general algorithm idea. We reproduced the CAAP study on both versions of the OpenMS algorithm, and unfortunately, the current version of OpenMS no longer achieves the results declared in the original study, achieving significantly worse results. We added a paragraph with this information in Suppl. Mat., Section 4, as below:

'OpenMS alignment algorithm performed best in the CAAP study. Originally, the authors of this study evaluated the OpenMS version 1.0. Its alignment algorithm was reimplemented in 2012 and the previous version is no longer bundled with the OpenMS package. We reproduced the evaluation of the CAAP study on the current version of OpenMS. Unfortunately, the current alignment algorithm is achieving significantly worse results despite strenuous attempts to adjust the algorithm parameters to the data. Its alignment precision and recall are on average 60 percentage points lower than the results reported in the CAAP study.'

> 7. For the biomarker analysis, could the authors indicate some kind of
> baseline expectations? It is now very hard for a reader to evaluate if these
> results are good.

Because this kind of benchmarking is not popular, there is no obvious choice for a baseline. For this reason, we repeated the same analysis with OpenMS, which, for this setup, is working well but only for technical repetitions of a single experiment (IR equal to 0.81, 0.76, 0.85, 0.83). For the experiment across multiple BaP concentrations, its IR falls to 0.75.

> Minor
>
> 1. I think it is a bit much to call retention time alignment "One of the most
> important challenges of the automated LC-MS analysis is the correction of
> errors cause by retention time (RT) drift.". Indeed, the problem is important,
> but one of the most important? There are a lot of open problems in proteomics,
> both on the hardware and data analysis side.

We modified this statement as follows (+ a preceding sentence for readability):

'(...) designing algorithms for efficient and precise analysis of LC-MS datasets remains challenging.

One of these challenges is the correction of errors caused by retention time (RT) drift.'

> 2. The following sentence is grammatically incorrect and I think it does not
> add anything to the better comprehension of the subject or problem at hand:
> "Also, inaccuracies of the detector in mass spectrometer may result in variable
> mass-tocharge ratio (M/Z) measurements, but in comparison to RT, the required
> correction is of essentially lower order of magnitude.". I suggest to remove
> this sentence.

As suggested, we removed this sentence.

> 3. The following sentence is hard to understand, what do the authors mean? Can
> they rewrite the sentence, so it is easier to understand? "It may change the
> whole chromatogram variably from sample to sample."

We rewrote the entire paragraph, see Major 2.

> 4. What are "fragments of the chromatogram". I find a lot of the terminology
> used confusing. Also because the terms used are not common in proteomics
> (/LC-MS) field.

We rewrote the entire paragraph, see Major 2.

> 5. What do the authors mean with this statement? "For samples
> analyzed under the same conditions, alignment is required to correct errors,
> while for samples analyzed under different experimental conditions, alignment
> is required to discriminate the corresponding biomarkers significant for
> further analysis.". So error correction is only needed for the same conditions?
> What errors are the authors talking about? And how is the connection made
> between biomarkers and different experimental conditions? This sentence is
> nonsensical.

We removed this statement and focused on LFQ instead, see Major 1.

> 6. Please rewrite this sentence "It overcomes the limitations of currently
> existing algorithms and deals properly even with RT drift, which swaps signals
> elution order over samples.". Deals properly with what?

We rewrote this sentence as follows:

'It overcomes the limitations of currently existing algorithms and properly resolves the correspondence of analytes of swapped elution order.'

> 7. Reference 26 and 27 are duplicated… So I guess there is a little bit less
> recent interest in the optimal transport approach? ;)

References 26 and 27 (in the previous paper version, now 34, 35) are two different papers by the same authors describing different setups (the former describes the fully discrete case, the latter semi-discrete case).

Although OT theory has over 50 years of history, its modern approach dates back to 2013 when Marco Cuturi proposed OT computation with the Sinkhorn-Knopp algorithm [1]. The application of optimal transport in life sciences is a case of the last 3-4 years since Chizat et al. work in 2018 (reference 40), where they proposed a technique of approximating the transport plan (cf. [2] or [3]).

Accordingly, interest in applying OT in MS is indeed very recent. Papers, which we cited in this work (references 27, 32, 34, 35), are to the best of our knowledge the only works of applying OT in MS, except one older, preliminary work written by our group [4] and two another from 2021: one more theoretical [5], one for elution profiles [6]. We added [6] to references (33) to show a more broad interest in OT in MS.

[1] Cuturi, M. (2013). Sinkhorn distances: Lightspeed computation of optimal transport. Advances in neural information processing systems, 26.
[2] Cang, Z., & Nie, Q. (2020). Inferring spatial and signaling relationships between cells from single cell transcriptomic data. Nature communications, 11(1), 1-13.
[3] Demetci, P., Santorella, R., Sandstede, B., Noble, W. S., & Singh, R. (2022). SCOT: Single-cell multi-omics alignment with optimal transport. Journal of Computational Biology, 29(1), 3-18.
[4] Majewski, S., Ciach, M. A., Startek, M., Niemyska, W., Miasojedow, B., & Gambin, A. (2018). The wasserstein distance as a dissimilarity measure for mass spectra with application to spectral deconvolution. In 18th International Workshop on Algorithms in Bioinformatics (WABI 2018). Schloss Dagstuhl-Leibniz-Zentrum fuer Informatik.
[5] Moorthy, A. S., & Kearsley, A. J. (2021). Pattern similarity measures applied to mass spectra. In Progress in Industrial Mathematics: Success stories (pp. 43-53). Springer, Cham.
[6] Permiakova, O., Guibert, R., Kraut, A., Fortin, T., Hesse, A. M., & Burger, T. (2021). CHICKN: extraction of peptide chromatographic elution profiles from large scale mass spectrometry data by means of Wasserstein compressive hierarchical cluster analysis. BMC bioinformatics, 22(1), 1-30.

> 8. In the list of abbreviations it says "Alignment" instead of Alignment.

Corrected.

Reviewer 2

> Comments:
> Skoraczyński et al describe an algorithm for retention time alignment which
> makes use of the generalized Wasserstein distance. This is beneficial because
> it allows to also consider peak shape and isotopic distribution to find
> corresponding features in MS runs. Based on several benchmark data sets the
> authors demonstrate comparable performance as achieved by the best algorithms
> currently available.
> The article is well written, and the authors managed to find an excellent
> balance for mathematical detail presented which makes the article good to read
> despite the complicated subject.
>
> Major Comments:
> - The biggest data set the authors use in their benchmarks is the CAAP P2
> data set which comprises 5 fractions run in triplicates. Precision achieved
> here is much lower than in the smaller P1 data set. In the discussion the
> authors point out that their algorithm has difficulties with distant features.
> Obviously, retention time deviations tend to increase in larger studies. To
> evaluate if this might be an issue constraining the performance of the
> presented algorithm, the authors should also benchmark on a larger data set.
> This will also be of high interest in times where the size of proteomics
> studies continuously grows.

The precision drop from duplicate P1 set to triplicate P2 set is observable for all algorithms. It is because finding the feature correspondence for two sets is relatively easy (e.g. MassUntangler is implemented only for pairwise alignment, Alignstein has a special, simplified implementation for 2 chromatograms). However, finding feature matching for more than two chromatograms is significantly more difficult, both for conceptual (So what formal problem we are solving?) and implementation sides (and how to do it?).

To benchmark on a larger dataset, we added the description of benchmarking on the M1 dataset from the CAAP study. It consists of 44 metabolomic chromatograms. Initially, we focused on proteomics and omitted this set due to its data dump incompleteness. Based on data provided by the authors, the

evaluation script would not give a clear answer if Alignstein performs better or worse than the other tools. Now, we resolved this obstacle by detecting features de novo. We used the tool and parameters from the original study (XCMS, method = "centWave", peakwidth = c(20, 50), snthresh = 5, ppm = 12). We matched newly detected features with the original ones and ran the evaluation script for such matched features. Results are competitive (precision 0.91, recall 0.88, F-score 0.89), which shows not only good scalability but also that Alignstein is not limited to proteomics. We reported the results in Table 1. Feature de novo detection is described in Suppl. Mat. section 4:

'M1 dataset lacks the spatial information of analyzed features. For this reason, we reproduced feature detection using XCMS3 with parameters detailed in the CAAP study. We matched features from the study with newly generated features by checking if the previous feature representation falls within the bounding box of new features. Such matched features were further input of the evaluation script.'

> Minor Comments:
> - The authors should mention the run time of the algorithm on the individual
> benchmark data sets and the computer on which the benchmarks were performed.

Good remark, we measured runtimes and presented results in Table 2. We added the following details of the computer in the Methods sections:

'Computation was done on a computer with Linux operating system and 24 Intel Xeon E5-2620 2.10 GHz processors + 62 GB RAM. We measured wall time using Linux built-in time command.'

> - Figure 1 in Box Clustering: hierarchical *clustering*

Corrected.

> - Page 7 after formula (1): where d(x, y) is *a distant* between peaks x and
> y; should be *the distance*

Corrected.

> - Page 8: We used input chromatograms as mzML and mzXML files and features as
> featureXML files provided by authors, as well as *their?* alignment precision
> and alignment recall evaluation script

We rewrote this sentence as follows:

'We used input chromatograms as mzML and mzXML files and features as featureXML files provided by authors of the CAAP study. We measured alignment precision and recall using an evaluation script written in R programming language by the authors of this study.'

> - Methods: as the paper deals with retention time alignment, the authors
> should also provide LC parameters used in the sample data sets (precolumn?,
> solvents, gradient, column, flow rate)

We added the following information to 'Mussels toxicological response experiment summary' Section in Methods:

'Acclaim PepMap C18 nano column 75 µm × 25 cm, 3 µm, 100 Å plus bypass, a linear gradient of 96% buffer A (0.5% Acetic Acid) and 4% buffer B (80% acetonitrile in 0.5% acetic acid) to 60% buffer A and 40% buffer B, a flow rate of 300 ml/min for 120 minutes).'

Close
