# Supplementary material for: Alignstein: Optimal transport for improved LC-MS retention time alignment
Source: Gigascience. 2022 Nov 3;11:giac101. doi: 10.1093/gigascience/giac101 (PMC9633278; doi:10.1093/gigascience/giac101)
Supplement: giac101_Response_to_Reviewer_Comments_Revision_1 [file giac101_response_to_reviewer_comments_revision_1.pdf]

Author's Response To Reviewer Comments

Close

Dear Editor,

We have made all the formatting changes you requested.

Grzegorz Skoraczyński, Anna Gambin, Błażej Miasojedow

Close
