# Supplementary material for: Alignstein: Optimal transport for improved LC-MS retention time alignment
Source: Gigascience. 2022 Nov 3;11:giac101. doi: 10.1093/gigascience/giac101 (PMC9633278; doi:10.1093/gigascience/giac101)
Supplement: giac101_Reviewer_1_Report_Original_Submission — Robbin Bouwmeester -- 6/24/2022 Reviewed [file giac101_reviewer_1_report_original_submission.pdf]

Reviewer Report

Title: Alignstein: optimal transport for improved LC-MS retention time alignment

Version: Original Submission **Date:** 6/24/2022

Reviewer name: Robbin Bouwmeester

Reviewer Comments to Author:

The manuscript presents a new algorithm for retention time alignment in LC-MS data. The algorithm called alignstein uses a more appropriate multi-dimensional alignment strategy than existing algorithms. The authors should however investigate more of the available methods that try to achieve a similar goal (e.g., quandenser). In addition to this the introduction should be rewritten a bit to make it comprehensible. In the results the authors did not convince me of the better performance of alignstein. I admit that this might be due to the available data or benchmarks, but if this is the case I would have at least expected a discussion about these limitations. I suggest a major revision as I think the authors are likely able to address all my concerns.

Major

1. Why is this needed? And what statistical analysis are you talking about? Do you mean quantification? In my opinion that is not a statistical analysis. "RT drift needs to be corrected prior to statistical LC-MS analysis."
2. The authors mention "Typically, it is caused by external conditions of variable nature, such as temperature [7].", but before it was mentioned it is hard to control experimental variables to counter retention time drift. External temperature is not hard to control. You can have chamber heating, pre-column heating, and even post-column heating.
3. I think the authors should have a look at "Quandenser" as it resolves a lot of the issues raised. The, M., KÃ¤ll, L. Focus on the spectra that matter by clustering of quantification data in shotgun proteomics. Nat Commun 11, 3234 (2020). https://doi.org/10.1038/s41467-020-17037-3
4. I would recommend the authors to look more into the literature between quantification and retention time alignment. The manuscript in its current form does not speak of quantification. While most people apply retention time alignment to better quantification. Also, the authors would have good benchmarks to test the performance of their algorithm through the use of these controlled experiments. E.g., see: Dowell, J. A., Wright, L. J., Armstrong, E. A., & Denu, J. M. (2021). Benchmarking quantitative performance in label-free proteomics. ACS omega, 6(4), 2494-2504.
5. I am very skeptical of evaluating performance on swapped signal with simulated data. I would urge the authors to seek further validation that does not involve simulated data. Or at least explain in detail why this is not possible to perform on experimentally acquired data.
6. Differences between Alignstein and OpenMS seem relatively small, if they perform equally is there another reason to use alignstein? Why is this difference so small when alignstein uses a much more sophisticated (according to the authors) algorithm for retention time alignment? Is the benchmark not suitable for evaluating?
7. For the biomarker analysis, could the authors indicate some kind of baseline expectations? It is now

very hard for a reader to evaluate if these results are good.
Minor
1. I think it is a bit much to call retention time alignment "One of the most important challenges of the automated LC-MS analysis is the correction of errors cause by retention time (RT) drift.". Indeed, the problem is important, but one of the most important? There are a lot of open problems in proteomics, both on the hardware and data analysis side.
2. The following sentence is grammatically incorrect and I think it does not add anything to the better comprehension of the subject or problem at hand: "Also, inaccuracies of the detector in mass spectrometer may result in variable mass-tocharge ratio (M/Z) measurements, but in comparison to RT, the required correction is of essentially lower order of magnitude.". I suggest to remove this sentence.
3. The following sentence is hard to understand, what do the authors mean? Can they rewrite the sentence, so it is easier to understand? "It may change the whole chromatogram variably from sample to sample."
4. What are "fragments of the chromatogram". I find a lot of the terminology used confusing. Also because the terms used are not common in proteomics (/LC-MS) field.
5. What do the authors mean with this statement? "For samples analyzed under the same conditions, alignment is required to correct errors, while for samples analyzed under different experimental conditions, alignment is required to discriminate the corresponding biomarkers significant for further analysis.". So error correction is only needed for the same conditions? What errors are the authors talking about? And how is the connection made between biomarkers and different experimental conditions? This sentence is nonsensical.
6. Please rewrite this sentence "It overcomes the limitations of currently existing algorithms and deals properly even with RT drift, which swaps signals elution order over samples.". Deals properly with what?
7. Reference 26 and 27 are duplicated… So I guess there is a little bit less recent interest in the optimal transport approach? ;)
8. In the list of abbreviations it says "Alignment" instead of Alignment.

Level of Interest

Please indicate how interesting you found the manuscript: Choose an item.

Quality of Written English

Please indicate the quality of language in the manuscript: Choose an item.

Declaration of Competing Interests

Please complete a declaration of competing interests, considering the following questions:

- Have you in the past five years received reimbursements, fees, funding, or salary from an organisation that may in any way gain or lose financially from the publication of this manuscript, either now or in the future?
- Do you hold any stocks or shares in an organisation that may in any way gain or lose financially from the publication of this manuscript, either now or in the future?
- Do you hold or are you currently applying for any patents relating to the content of the manuscript?
- Have you received reimbursements, fees, funding, or salary from an organization that holds or has applied for patents relating to the content of the manuscript?
- Do you have any other financial competing interests?
- Do you have any non-financial competing interests in relation to this paper?

If you can answer no to all of the above, write 'I declare that I have no competing interests' below. If your reply is yes to any, please give details below.

I declare that I have no competing interests

I agree to the open peer review policy of the journal. I understand that my name will be included on my report to the authors and, if the manuscript is accepted for publication, my named report including any attachments I upload will be posted on the website along with the authors' responses. I agree for my report to be made available under an Open Access Creative Commons CC-BY license (http://creativecommons.org/licenses/by/4.0/). I understand that any comments which I do not wish to be included in my named report can be included as confidential comments to the editors, which will not be published.

Choose an item.

To further support our reviewers, we have joined with Publons, where you can gain additional credit to further highlight your hard work (see: https://publons.com/journal/530/gigascience). On publication of this paper, your review will be automatically added to Publons, you can then choose whether or not to claim your Publons credit. I understand this statement.

Yes Choose an item.
