# Supplementary material for: Alignstein: Optimal transport for improved LC-MS retention time alignment
Source: Gigascience. 2022 Nov 3;11:giac101. doi: 10.1093/gigascience/giac101 (PMC9633278; doi:10.1093/gigascience/giac101)
Supplement: giac101_Reviewer_2_Report_Original_Submission — Karl Mechtler -- 7/29/2022 Reviewed [file giac101_reviewer_2_report_original_submission.pdf]

Reviewer Report

Title: Alignstein: optimal transport for improved LC-MS retention time alignment

Version: Original Submission **Date:** 7/29/2022

Reviewer name: Karl Mechtler

Reviewer Comments to Author:

Comments:
SkoraczyÅ„ski et al describe an algorithm for retention time alignment which makes use of the generalized Wasserstein distance. This is beneficial because it allows to also consider peak shape and isotopic distribution to find corresponding features in MS runs.
Based on several benchmark data sets the authors demonstrate comparable performance as achieved by the best algorithms currently available.
The article is well written, and the authors managed to find an excellent balance for mathematical detail presented which makes the article good to read despite the complicated subject.
Major Comments:
- The biggest data set the authors use in their benchmarks is the CAAP P2 data set which comprises 5 fractions run in triplicates. Precision achieved here is much lower than in the smaller P1 data set. In the discussion the authors point out that their algorithm has difficulties with distant features. Obviously, retention time deviations tend to increase in larger studies. To evaluate if this might be an issue constraining the performance of the presented algorithm, the authors should also benchmark on a larger data set. This will also be of high interest in times where the size of proteomics studies continuously grows.
Minor Comments:
- The authors should mention the run time of the algorithm on the individual benchmark data sets and the computer on which the benchmarks were performed.
- Figure 1 in Box Clustering: hierarchical *clustering*
- Page 7 after formula (1): where d(x, y) is *a distant* between peaks x and y; should be *the distance*
- Page 8: We used input chromatograms as mzML and mzXML files and features as featureXML files provided by authors, as well as *their?* alignment precision and alignment recall evaluation script
- Methods: as the paper deals with retention time alignment, the authors should also provide LC parameters used in the sample data sets (precolumn?, solvents, gradient, column, flow rate)

Level of Interest

Please indicate how interesting you found the manuscript: Choose an item.

Quality of Written English

Please indicate the quality of language in the manuscript: Choose an item.

Declaration of Competing Interests

Please complete a declaration of competing interests, considering the following questions:

- Have you in the past five years received reimbursements, fees, funding, or salary from an organisation that may in any way gain or lose financially from the publication of this manuscript, either now or in the future?
- Do you hold any stocks or shares in an organisation that may in any way gain or lose financially from the publication of this manuscript, either now or in the future?
- Do you hold or are you currently applying for any patents relating to the content of the manuscript?
- Have you received reimbursements, fees, funding, or salary from an organization that holds or has applied for patents relating to the content of the manuscript?
- Do you have any other financial competing interests?
- Do you have any non-financial competing interests in relation to this paper?

If you can answer no to all of the above, write 'I declare that I have no competing interests' below. If your reply is yes to any, please give details below.

I declare that I have no competing interests

I agree to the open peer review policy of the journal. I understand that my name will be included on my report to the authors and, if the manuscript is accepted for publication, my named report including any attachments I upload will be posted on the website along with the authors' responses. I agree for my report to be made available under an Open Access Creative Commons CC-BY license (http://creativecommons.org/licenses/by/4.0/). I understand that any comments which I do not wish to be included in my named report can be included as confidential comments to the editors, which will not be published.

Choose an item.

To further support our reviewers, we have joined with Publons, where you can gain additional credit to further highlight your hard work (see: https://publons.com/journal/530/gigascience). On publication of this paper, your review will be automatically added to Publons, you can then choose whether or not to claim your Publons credit. I understand this statement.

Yes Choose an item.
